# Cardiovascular Effects of Chocolate and Wine—Narrative Review

**DOI:** 10.3390/nu13124269

**Published:** 2021-11-26

**Authors:** Beata Sperkowska, Joanna Murawska, Anna Przybylska, Marcin Gackowski, Stefan Kruszewski, Maciej Durmowicz, Dorota Rutkowska

**Affiliations:** 1 Department of Toxicology and Bromatology, Faculty of Pharmacy, L. Rydygier Collegium Medicum in Bydgoszcz, Nicolaus Copernicus University in Torun, A. Jurasza 2 Street, PL-85089 Bydgoszcz, Poland; joanna.murawska94@wp.pl (J.M.); aniacm@cm.umk.pl (A.P.); marcin.gackowski@cm.umk.pl (M.G.); 2 Biophysics Department, Faculty of Pharmacy, L. Rydygier Collegium Medicum in Bydgoszcz, Nicolaus Copernicus University in Torun, Jagiellońska 13 Street, PL-85067 Bydgoszcz, Poland; skrusz@cm.umk.pl; 3 Department of Physiotherapy, Faculty of Health Sciences, L. Rydygier Collegium Medicum in Bydgoszcz, Nicolaus Copernicus University in Torun, Techników 3 Street, PL-85094 Bydgoszcz, Poland; maciej.durmowicz@cm.umk.pl; 4 Laboratory for Medical Education, Faculty of Medicine, L. Rydygier Collegium Medicum in Bydgoszcz, Nicolaus Copernicus University in Torun, St. Florian’s 12 Street, PL-85030 Bydgoszcz, Poland; dorota.rutkowska@cm.umk.pl

**Keywords:** bioactive compounds, chocolate, wine, cardiovascular benefits, cardiovascular risk, drug–food interactions, inflammation, atherosclerosis, oxidative stress

## Abstract

The consumption of food for pleasure is mainly associated with adverse health effects. This review was carried out to verify recent reports on the impact of chocolate and wine consumption on cardiovascular health, with a particular focus on atherosclerosis. On one side, these products have proven adverse effects on the cardiovascular system, but on the other hand, if consumed in optimal amounts, they have cardiovascular benefits. The submitted data suggest that the beneficial doses are 30–50 g and 130/250 mL for chocolate and wine, respectively, for women and men. The accumulated evidence indicates that the active ingredients in the products under consideration in this review are phenolic compounds, characterized by anti-inflammatory, antioxidant, and antiplatelet properties. However, there are also some reports of cardioprotective properties of other compounds such as esters, amines, biogenic amines, amino acids, fatty acids, mineral ingredients, and vitamins. Our narrative review has shown that in meta-analyses of intervention studies, consumption of chocolate and wine was positively associated with the beneficial outcomes associated with the cardiovascular system. In contrast, the assessment with the GRADE (Grading of Recommendations Assessment, Development and Evaluation) scale did not confirm this phenomenon. In addition, mechanisms of action of bioactive compounds present in chocolate and wine depend on some factors, such as age, sex, body weight, and the presence of additional medical conditions. Patients using cardiovascular drugs simultaneously with both products should be alert to the risk of pharmacologically relevant interactions during their use. Our narrative review leads to the conclusion that there is abundant evidence to prove the beneficial impact of consuming both products on cardiovascular health, however some evidence still remains controversial. Many authors of studies included in this review postulated that well-designed, longitudinal studies should be performed to determine the effects of these products and their components on atherosclerosis and other CVD (Cardiovascular Disease) disease.

## 1. Introduction

Presently, cardiovascular diseases occupy the infamous number one among causes of death worldwide. In 2019, the latest year for which global statistics have already been calculated, nearly 18.6 million people died. That marks a 17.1 percent rise over the last decade. Moreover, the number of cases of CVDs in 2019 was as high as 523.3 million, thus an increase of 26.6 per cent was observed between 2010 and 2019 [1]. Cardiovascular diseases (CVDs) is a general term for disorders related to the heart and circulatory system. They include nearly one hundred disease units, classified as I00 up to I99 in the International Statistical Classification of Diseases and Health Problems (ICD-10) [2]. Available data indicate that atherosclerosis (I70) is the highest risk, and its complications of ischemic heart disease (I25) and stroke (I63) were the most frequent causes of mortality in 2019 [2]. Atherosclerosis affects the populations of well-developed countries. Oxidative stress and inflammatory conditions are two main factors affecting the development of atherosclerosis and cardiovascular diseases [3,4,5,6]. A crucial factor in the pathogenesis of formation and development of atherosclerotic plaque is the accumulation of oxidized low-density lipoproteins (LDL), foam cells, and extracellular aggregates of cholesterol between the endothelium and muscle layer of the vessels [7,8]. Modifications of LDL structure and vascular endothelium damages occur with the participation of free radicals [9,10]. Therefore, the application of food and beverages containing various antioxidative compounds can be helpful in prophylaxis and the treatment of atherosclerosis and other cardiovascular diseases. The beneficial flavonoid effect is the result of their ability to reduce the excess of free radicals as well as to prevent their formation inside the body cells [3,11,12]. It is possible by inhibition of the oxidative stress factors. By free radical reduction, the antioxidants protect the LDL lipoprotein structures among other things against oxidation and also inflammation, which is responsible for atherosclerosis development, but this hypothesis has many opponents in the research community [1,2].

Risk factors for CVDs include smoking, diabetes, hypertension, abnormalities in serum total cholesterol and cholesterol fractions, overweight or obesity, family history of early CVD, and physical inactivity [3,4].

Various papers underline the cardiovascular outcomes of healthy and unhealthy dietary patterns [5,6,7,8,9,10,11,12,13,14]. Moreover, recently, much attention has been paid to the Mediterranean Diet (MedDiet) and the DASH diet. These healthy eating plans are beneficial to human health and have an established role in CVD prevention [15]. The traditional MedDiet is based on a high consumption of plant foods, fresh fruit, and olive oil, a moderate intake of fish and poultry, a low intake of dairy products, red meat, processed meats, and sweets, and a low-to-moderate alcohol intake. However, alcohol intake on the MedDiet is a subject of controversy. Some studies have shown that alcohol is a main risk factor of premature morbidity and mortality [16,17,18]. Numerous studies indicated that both chocolate and wine are rich sources of bioactive compounds such as polyphenols, or others but in smaller amounts, such unsaturated fatty acids, amino acids, amines, vitamins, minerals, etc. determined its cardioprotective effects [19,20,21,22,23]. Since the French paradox, much evidence has been published of a beneficial link between drinking alcohol and cardiovascular health. In a published meta-analysis involving 48,423 men and women, the conclusions showed that for alcohol consumption with all assessed J-shaped outcomes compared with non-drinkers, risk reduction peaked at 7 g/day (relative risk 0. 79, 95% CI 0.73–0.85) for all-cause mortality, 8 g/day (0.73, 0.64–0.83) for cardiovascular mortality, and 6 g/day (0.50, 0.26–0.96) for cardiovascular events, and remained significant up to 62, 50, and 15 g/day, respectively [17].

Furthermore, the continuous evolvement of SARS-CoV-2 pandemic affects CVDs incidence. In this light, it is predicted that the global burden of cardiovascular disease will rise exponentially in the nearest future [24,25,26,27,28,29,30]. The increase in heart disease will be determined not only by the direct damage to the heart muscle caused by SARS-CoV-2 but also by the consumption of more products such as wine and chocolate, which provide significant amounts of energy. According to some authors, the consumption of abovementioned products increased during COVID-19 pandemic [31,32]. It is known that high intakes of energy, alcohol, fat, and simple carbohydrates are associated with obesity and other non-communicable diseases [33,34,35,36,37].

It is also important that patients treated for cardiovascular diseases should be informed by their doctors about the risk of interactions between the medication they take and the products they consume such as chocolate and wine. Although drug–food interactions are considered crucial for the efficacy of treatment in various diseases, the mechanisms of many interactions of CVD and anti-atherosclerotic drugs (antiplatelet drugs, statins fibrates, PCSK9 (proprotein convertase subtilisin/kexin type 9) inhibitors, cholesterol absorption inhibitors, or nicotinic acid) with chocolate and wine have not yet been studied. Little is currently known about the interaction of wine with statins and antiplatelet drugs [1,38,39].

When acetylsalicylic acid is taken together with wine, there is a synergistic interaction and enhancement of the antiplatelet effect due to inhibition of cyclooxygenase I (COX-I) in platelets, with consequent bleeding [40,41]. On the other hand, the combined use of statins (e.g., simvastatin, atorvastatin) with alcohol leads to an inhibition of CYP450 3A4-mediated metabolism of these drugs and an increase in their serum concentration as a result of shared hepatic metabolism [39].

However, interactions may also occur with other components of these products, especially polyphenols, and within this group of compounds, drug interactions with resveratrol, followed by quercetin, have been described most extensively. The results of studies on interactions between aspirin and gallic acid found in wine are also available [38]. It should be emphasized that the pharmacokinetics and pharmacodynamics of drugs can be altered by the presence of macronutrients such as fat [42]. Thus, lipophilic drugs are better absorbed in the presence of fat; thus, simvastatin, atorvastatin, and lovastatin are likely to be better absorbed after consuming chocolate, whereas fluvastatin and pravastatin, due to their hydrophilic nature, in the presence of wine [43]. Another point to note is the effect of the pH of the food. Wine with a pH of 3.5–4.0 causes acidification of the gastrointestinal contents, which reduces the absorption of alkaline drugs and increases the absorption of acidic drugs [44].

The current narrative review was conducted to verify recent reports on the beneficial and adverse effects of “pleasure foods” consumed for health heart.

## 2. Materials and Methods

The methodology of the current narrative review was based on searching the PubMed^®^, Web of Science TM, and Scopus databases. The search was conducted from March 2021 to September 2021 with the use of following keywords: chocolate, wine, alcohol, atherosclerosis, bioactive compounds, cardiovascular benefits, cardiovascular risk, cholesterol, inflammation, inflammatory markers, oxidative stress, vascular function, phytochemicals, polyphenols, and drug–food interactions. Exclusion criteria were: a) intervention studies published before 2010; b) articles not focused on the effects of chocolate and wine on CVD status; and c) interventions performed ex vivo or in silico. The literature incorporated in this review includes meta-analyses, experimental and clinical studies, and references of identified papers but excludes books, letters, editorials, conference abstracts, and commentaries. This narrative review is a recapitulation of the evidence of the last 11 years (2010–2021) moved into clinical practice. In order to avoid omitting any important research article, reference lists of each article were manually searched. Authors selected all studies in English, available in human and animal studies, in vivo and in vitro studies, with a primary focus on the development and progression of atherosclerosis. Notably, authors focused on the controversial but probable benefits of the chocolate and alcohol beverages components flavonoids in order to examine their general impact on cardiovascular risk factor intermediates and cardiovascular endpoints. Moreover, the authors underlined that cardiology patients who simultaneously ingest medicines and consume chocolate and wine are exposed to drug–food interactions. In this work, authors decided to conduct a narrative review instead of traditional systematic review, mainly because of too little consistent evidence about this topic.

## 3. Atherosclerosis

Atherosclerosis, a condition characterized by the deposition of atherosclerotic plaques due to the accumulation of low-density lipoproteins [45,46] and fibrous substances in damaged arteries, is a major cause of ischemic heart disease (I25) and stroke (I63), which were the most common causes of mortality in 2019 [2,47]. It is well known that a chronic mild inflammatory reaction contributes to lipid accumulation occurring in the arterial walls in different locations of the circulatory system in the shape of atheroma plaques [48,49]. In healthy volunteers, a significant reduction in vascular cellular adhesion associated with VCAM-1 (vascular cell adhesion molecule 1) [50], intercellular adhesion molecule-1(ICAM-1) [51], IL-1a, and very late lymphocyte expression associated with very late activation antigen 4 (VLA-4) antigen was observed after red wine consumption [52].

Furthermore, phenolic compounds and red wine alcohol decreased CD40 antigen [53], CD40 ligand (cluster of differentiation 40), and monocyte chemoprotein expression [54]. Given the results of clinical studies, it is possible to ascertain the health-promoting effects of both chocolate [55,56] and wine [57,58,59] when consumed in moderate amounts and used prophylactically. In the absence of these studies, as argued by many investigators, the results of epidemiological studies cannot be considered definitive, especially after observing the striking differences between the outcomes of epidemiological and intervention studies evaluating, for instance, the efficacy of antioxidant vitamins on the cardiovascular system [60].

Empirical findings suggest that dietary polyphenols, especially flavonoids, play an integral role in ameliorating atherosclerosis, and their pleiotropic antioxidant and anti-inflammatory activities appear to antagonize atherogenesis [61,62].

Some observations suggest that chocolate and wine have cardioprotective potential in humans, as do other natural and unprocessed products (fish, olive oil, fruit, vegetables, and whole grains) that form the basis of the Mediterranean diet recommended by scientific cardiological societies [63].

Although the chemical components of chocolate and wine are different, similar beneficial effects have been observed for both products. It is worth pointing out that red wine, while having a good reputation, does not contain the cardioprotective flavan-3-ol in large concentrations, as is the case with the chocolate [64,65,66].

### 3.1. Chocolate

Due to its unique texture and taste, chocolate is a food that is usually reached for for pleasure. Still, for several years, it has been reconsidered as a source of beneficial bioactive compounds [20,37,46,65,67,68,69,70,71,72,73,74]. It is a confectionery product mainly made from cocoa beans (cocoa pulp), cocoa fat, and sugar. It may contain further ingredients, e.g., milk, nuts, alcohol, coffee, fruit, flavors. Directive 2000/36/EC of the European Union requires chocolate to contain not less than 35% of dry cocoa solids. Cocoa, the primary constituent of chocolate, contains a relevant amount of fat 30–50%, with approximately 33% oleic acid, 25% palmitic acid, and 33% stearic acid [75]. Polyphenols are also important ingredients of chocolate, which can make up to 10% of a whole bean’s dry weight [76,77].

Dark chocolate energy content is estimated at 515–593 kcal/100 g of product [77,78]. In addition to macronutrients, which are 40–50% cocoa butter (mostly saturated fats: stearic and palmitic, and unsaturated fats: oleic and linoleic), 53–64% carbohydrates, and protein 5–10%, chocolate is a product recommended as a valuable source of antioxidants, vitamins, and minerals, including magnesium, manganese, iron, phosphorus, zinc, selenium, potassium, biogenic amines, and amino acids [77]. An experimental study evaluating the fatty acid profile of Brazilian chocolates reported that they were a source of saturated and unsaturated fatty acids. Saturated palmitic and stearic acids were found at 0.9 and 203.78 mg/g, respectively, while unsaturated oleic and linoleic acids were 304 and 27.79 mg/g cocoa fat. The chemical composition of the chocolate was as follows: proteins 8.99%, fats 36.21%, carbohydrates 51.7%, ashes 1.7%, and moisture 1.3% [79]. Although chocolate is not classified as a good source of vitamins, recently, Kühn et al. [80] found that dark chocolate had from 1.90 to 5.48 µg/100 g vitamin D2, while white chocolate had lower vitamin D2 ranging from 0.19 to 1.91 µg/100 g. By taking an average vitamin D2 content of 2.3 µg/100 g of chocolate, it may be calculated that the everyday intake of vitamin D2 from chocolate in the German population was 0.5 µg [80,81].

Dala-Paula et al. [82] in their research found that chocolate with 70% cocoa was a good source of bioactive amines, mainly polyamines but also cadaverine, putrescine, phenylethylamine, tyramine, and tryptamine. Importantly, dark chocolate protein is a good source of histidine isoleucine, phenylalanine + tyrosine, and tryptophan but limiting for leucine, lysine, and threonine [82]. The nutritional values of the phenolic compounds of both chocolate and wine are shown in Table 1.

The most important cardioprotective ingredients as documented in the study are flavonoids from cocoa, including flavones, isoflavones, flavanones, flavonols, flavanols, and anthocyanins, which may improve cholesterol efflux capacity in vitro [83,84,85]. The average values of phenolic compounds in chocolate are shown in Table 2.

#### 3.1.1. Cardioprotective Properties of Chocolate

Cocoa flavanols’ protective CVD effects in dark chocolate have been the subject of many epidemiological and interventional trials [77,87,88]. Data from recent epidemiological studies support an association between high consumption of cocoa products rich in flavanols and a reduced incidence of CVD events, especially stroke and ischemic heart disease. Cocoa flavanols and flavan-3-ols are the main compounds responsible for this effect [89].

##### Epidemiologic Studies

Assessment of the impact of chocolate consumption on CVD and death risk was carried out using seven epidemiological studies published between 2011 and 2018 and demonstrated in Table 3. Across the seven studies, four participants completed food frequency questionnaires [87,90,91,92]. The research follows time ranging from 12.9 to 30 years and indicates a preventive effect of frequent chocolate intake (45% risk reduction compared to no consumption, *p* = 0.005). Additionally, chocolate intake was protective against non-fatal stroke events.

Buitrago-Lopez et al., showed in their study that the risk of stroke and ischemic heart disease was reduced by 29 and 37%, respectively, as a result of dark chocolate consumption. [93]. In addition, a study by Kwok et al. [67] found a reduction in the risk of stroke, in this case by 23%. In contrast, the risk of coronary atherosclerosis was reduced by 11% due to dark chocolate consumption [67]. In turn, a study by Larsson et al. [92] demonstrated a decrease in myocardial infarction (MI) risk in individuals consuming chocolate [92].

Data from two SRMAs observational studies examined the relationship between eating chocolate and cardiovascular risks [43,44]. Mentioned studies reveal that high chocolate intake considerably reduced the risk of coronary heart disease (pooled RRs ranging from 0.71 (95%CI: 0.56–0.92) to 0.90 (95%CI: 0.82–0.97)), stroke (pooled RRs ranging from 0.79 (95%CI: 0.70–0.87) to 0.84 (95%CI: 0.78–0.90), and cardiovascular mortality (pooled RR = 0.55 (95%CI: 0.36–0.83) [97].

The results of a large population-based prospective cohort study suggest that moderate chocolate consumption may have an inverse association with the risk of atrial fibrillation or flutter, although the influence of other factors should also be considered. As evidenced by Crichton et al. [96], consuming large amounts of chocolate and products rich in cocoa flavonols reduces the risk of type 2 diabetes predisposing to cardiovascular disease [68,96,98]. Yuan et al., in a meta-analysis of 14 prospective studies, concluded that chocolate consumption of six servings per week is responsible for reducing the risk of diabetes and coronary heart disease and may also prevent stroke [99].

Although chocolate consumption may have beneficial effects on cardiovascular health, there is still limited data evidence from large, prospective cohort studies [87]. During a median follow-up of 12.9 years, 3558 incident strokes cases (2146 cerebral infarctions and 1396 hemorrhagic strokes) were identified. After adjustment for age, body mass index, lifestyles, dietary intakes, and other risk factors, chocolate consumption was associated with a significantly lower risk of stroke in women (HR = 0.84; 95% CI, 0.71–0.99). However, the association in men was not significant (HR = 0.94; 95% CI, 0.80–1.10) [87].

In addition, in light of the evident relationship between serum cholesterol and cardiovascular risks, present evidence concentrates on dietary fats, in contrast to previous inconsistent focus on cardiovascular prevention due to dietary fat intake [97].

Veronese et al. [68], in their umbrella review, pointed out for the first time that the supporting evidence suggesting the benefits of chocolate consumption was weak. With more than one million participants, four studies found an association of chocolate consumption with reduced risk of cardiovascular disease, six with acute myocardial infarction, five with stroke, and a further six with diabetes. However, as the authors of the review pointed out, these studies were characterized by weak evidence of reliability. In contrast, the meta-analyses of intervention studies discussed in the same paper chocolate had a positive effect on vasodilation for three studies and two others on markers of insulin resistance. However, using the GRADE score, the evidence for these outcomes was not satisfactory [68].

As the effect size is much more prominent in clinical trials than prospective studies, this should be considered in formulating guidelines. Moreover, promising results from short-term clinical trials cannot be treated uncritically, as they are not always clinically significant and therefore may not represent a potential therapeutic option. Nevertheless, the combined results of this review suggest that chocolate is not simply a harmful food consumed for pleasure and should not be avoided for this reason [100].

##### Clinical Trials

Chocolate, in general, is not seen as a food with cardioprotective properties as is the case with red wine, thanks to the phenomenon of the French paradox [101]. However, studies conducted on the composition of this product show that it is in many ways superior to wine in its cardioprotective properties [102]. Scientists showed that flavone-3-ols found in cacao and dark chocolate have a higher potency than the stenols found in wine, as previously mentioned [103].

Unfortunately, this effect is probably counteracted by the high fat content, especially saturated fat [104], although according to some researchers, these fats also have some potential [105].

Despite many controversies, ongoing randomized controlled trials over the past ten years have reported many hopeful results related to the cardioprotective properties of chocolate and its key ingredient cocoa, some of which are presented in Table 4.

Studies included in our review usually took the form of a dietary intervention and were carried out in groups of 20 to 136 participants. All studies were recorded at clinicaltrials.gov. About 70% of the study participants were healthy subjects [65,72,107,108,109,111,112], the others were non-alcoholic steatohepatitis patients [113] and the obese [84,110].

The authors concluded that 20–50 g of dark chocolate or the equivalent amount of epicatechins could have a wide range of effects on the human body. The researchers suggest that chocolate has anti-inflammatory effects [107] and antioxidant properties [109]. Furthermore, improved lipid profile [110,114], reduced levels of creatinine, lactate, some amino acids and their breakdown products in urine, and increased levels of pyruvate and 4-hydroxyphenylacetate, a phenolic compound of bacterial origin [65], improved vascular function by reducing pressure in the middle brachial artery and promoting vascular relaxation, thereby improving the fit of the arterial system to the left ventricle [111], and more cocoa polyphenols improving endothelial function by downregulating Nox2 in NASH patients [113] have been observed.

Another study found that cocoa products containing about 0.1 g of epicatechin can again reliably increase FMD, and doses of cocoa flavanols of about 0.9 g can lower blood pressure in certain individuals, however, if consumed over a long period. Vlachojannis et al. [89] reported that to provide 0.9 g of total flavanols, one needs to consume approximately 100 to 500 g of chocolate. However, to give a therapeutic dose of epicatechin, one needs to consume 0.05 to 0.2 g of chocolate. It, therefore, seems advisable to demand that chocolate products marketed for their purported health benefits declare the amount of total flavanols and epicatechin [89].

Similarly, the results of a meta-analysis of 18 studies involving 367 participants suggest an improvement in FMD after consumption of chocolate, cocoa drink, or a combination of both (1.17%, 95% CI: 0.76%, 1.57%). The high I2 score (83.3%) suggests that most of the variability between studies is due to heterogeneity rather than randomization. Studies with the chocolate intervention showed an improvement in FMD of 0.84% (CI 0.19% to 1.50%). In contrast, the cocoa drink intervention showed a mean improvement in FMD of 1.13% (CI 0.78% to 1.48%); notable studies with the chocolate intervention had higher heterogeneity than studies with the cocoa drink intervention (I2 = 81.6% vs. I2 = 63.7%) [69].

In order to demonstrate the therapeutic effect of chocolate, Bertolami et al. [115] compared the effects of ezetimibe and functional foods containing plant sterol esters on oxidative stress biomarkers. In this double-blind study, 37 statin-treated subjects with type 2 diabetes were randomly assigned to take ezetimibe 10 mg/day or consume chocolate containing 2.1 g of plant sterol esters for six weeks. They found that ezetimibe increased superoxide dismutase (SOD) and glutathione reductase (GR) activity, while plant sterols did not alter these biomarkers. Among patients treated with both plant sterols and ezetimibe, oxLDL-C levels were reduced by 20 and 21.1%, respectively. Furthermore, almost 50% of patients treated with plant sterols achieved the recommended LDL-C status of <70 mg/dL [115,116].

### 3.2. Wine

For hundreds or even thousands of years, people have been consuming wine for a variety of reasons. Some individuals cannot imagine their lunch or gala dinner without this drink [117,118]. It is worth emphasizing that benefits from moderate alcohol consumption are not restricted to taste sensations but have been extensively described by many researchers. For this reason, red wine intake has been associated with decreasing a risk for coronary heart disease (CHD) [117,118,119].

The volume of consumption demonstrates its popularity. World wine consumption in 2020 was estimated at 234 mln hL; noting that its consumption has decreased by 3% compared to 2019, it can be assumed that this is still a significant volume [120].

#### 3.2.1. Phytochemical Structure of Wine

Polyphenols, which are present in red wine, form a complex mixture of flavonoids (such as anthocyanins and flavan-3-ols) and nonflavonoids (such as resveratrol, cinnamates, and gallic acid). The most plentiful group of polyphenols makes it to 50% of total phenolic constituents and incorporates flavan-3-ols with polymeric procyanidins (condensed tannins) [121,122].

Wine, by definition, is an alcoholic beverage made from fermented fresh grapes or grape juice (must). It is a composite blend of several hundred compounds occurring in very low levels, which nevertheless play an essential role in its formation and quality. Overall, the mean percentages of the major wine components are: 86% water; 12% ethanol; 1% glycerol and polysaccharides, trace elements; 0.5% different types of acids; and 0.5% volatile compounds [123]. The nutritional value of wine, as well as of chocolate, is presented in Table 2.

The phytochemical characteristics of wine involve compounds as such organic acids (acetic, citric, lactic, malic, succinic, and tartaric), nitrogen, phenolic compounds, mineral components, biogenic amines, alcohol, and sulfur dioxide. The organic acid level defines the taste of the wine. The nitrogen is a key nutrient for yeast growth and is necessary for successful fermentation. The phenol content in wine (natural phenol and polyphenols) includes a large group of several hundred substances [124,125]. Flavonoids as well as nonflavonoid phenolic compounds have an established role in the protective effects of wine on the circulatory system [126,127]. Nevertheless, the most extensively studied stilbene resveratrol is considered as the most important nonflavonoid compound responsible for the health benefits of red wine [103,128], although in the last few years, research results have been published confirming the cardioprotective properties of compounds such as quercetin [23,129], catechins [130], or proanthocyanidins [103,125].

Tannins in wine are an essential ingredient responsible for determining the taste characteristics of wine. If the tannin content is too low, the taste is bland, whereas if the tannin concentration is too high, the wine is astringent, which is described by the term “tanning the mouth epithelium” [122]. In addition, they have chemopreventive effects and play a role as antioxidants in wine. Elements such as iron, calcium, magnesium, phosphorus, potassium, and manganese, which are present in wine in trace amounts, are responsible for these effects [131].

Hydroxytyrosol, which can be found primarily in olive oil and as some researchers have found, in red wine, also has strong antioxidant properties [132]. Furthermore, preclinical studies have linked OHTyr with anti-inflammatory, anti-proliferative, pro-apoptotic, antibacterial, and neuroprotective properties. In turn, clinical studies have shown that OHTyr-rich foods present in the Mediterranean diet (MedDiet) improve the lipid profile, protect against lipid oxidation, and prevent primary cardiovascular disease (CVD) [54]. Potent antioxidants include biogenic amines too, but one of them, tyramine, can lead to serious side effects such as the so-called cheese effect [133]. The ambivalent properties also characterize alcohol in wine (6–22%) as a natural product of alcoholic fermentation. Potentially dangerous compounds occurring in wine due to fermentation are sulfur compounds that occur naturally in wine, either as a result of the fermentation process or added artificially as a preservative and wine stabilizer [18]. Most of the compounds have focused on epidemiological and clinical studies on cardioprotective properties for more than 50 years.

#### 3.2.2. Cardioprotective Effects of Wine

Both clinical [97,121] and epidemiological [73,103,134] studies on wine’s positive health properties indicated that regular and moderate wine consumption (150 to 300 mL wine per day) is associated with reduced prevalence of cardiovascular disease [135], hypertension [18], diabetes [136], cancer [137], and even osteoporosis [138].

The studies presented in our narrative review of the cardiovascular effects of red wine focused on components such as alcohol and phenolic compounds with nonflavonoids compounds (resveratrol [54,139,140] and tyrosols [132]). The association of moderate alcohol consumption with incident cardiovascular deaths has been most extensively reported in the literature [117]. Many papers emphasize the U- and J-shaped nature of the association, indicating that abstainers are at higher risk of cardiovascular disease and death than drinking men [17,141]. Although this phenomenon has been studied since the French paradox, there are still areas that require well-designed, long-term randomized studies. Data from this narrative review show that alcohol correlates with better HDL results but does not affect other parameters such as TG, LDL, coagulation factors, CRP, and oxidized LDL levels [142,143].

##### Epidemiological Studies

In observational studies carried out over a decade ago, a protective effect of moderate alcohol drinking on the occurrence of cardiovascular events was shown, but the exact mechanisms involved are still not sufficiently known [144,145].

Many authors argue that modulation of circulating cholesterol is the best-established protective factor of alcohol intake [146,147,148] and inverse relations of HDL-cholesterol and CVD risk operate substantially via removal of lipid deposits in large blood vessels [149,150,151].

The thirteen observational studies examined in our narrative review were most often concerned with assessing the effect of alcohol on HDL levels (three studies) as well as mortality (four studies). They were usually cohort-based and randomized studies. In five trials, researchers used validated questionnaires on food intake, including wine or its constituents such as resveratrol or tyrosol. In the studies on the association of alcohol consumption and mortality, as in previous studies, it was shown that people who do not consume alcohol have a higher risk of death and cardiovascular incidents [146,152,153]. The study involved both healthy volunteers and patients with various cardiovascular conditions. Two of the observational studies looked at the relationship between people’s lifestyle (Mediterranean Diet and physical activity) with atherosclerosis and consumption [154,155]. In one study, the authors showed that LDL to HDL fractions improved in women and men who consumed 100 and 200 mL of wine, respectively [155]. A summary of epidemiological studies assessing the relationship between wine and other beverages consumption and CVD is set out in Table 5.

In a population-based cohort study (Moli-sani study) to determine the association between alcohol consumption and incident HF and AF, moderate alcohol consumption (10–20g/d) was shown to reduce the risk of heart failure (HF) but not AF compared with abstainers. The assessed biochemical parameters HDL and TC were related to alcohol consumption. Those consuming more than 48 g per day of alcohol had the highest HDL levels at 63 mg/dL, but total cholesterol levels were the highest in the entire study cohort at 226 mg/dL. Non-drinkers and occasional drinkers had levels of 54 and 207 mg/dL, respectively [156]. The authors found that the mean reduction in risk of total mortality was 11% and that alcohol consumption >20 g/day was related to a 13% reduction in risk of total mortality. Similar results were observed for deaths from cardiovascular (CV) reasons alone. The relationship of alcohol to mortality was similar in men and women. However, it was more pronounced in those who preferred wine, suggesting that the benefit may be due to other components rather than ethanol. Mediation analysis demonstrated that high density lipoprotein cholesterol explained 2.9 and 18.7% of the relation between low alcohol consumption and all-cause and cardiovascular mortality, respectively [156].

However, the findings of the GISSI-Prevenzione study indicate that moderate wine intake seems to be significantly associated with lower CVD incidence and total mortality compared with non-drinkers. In the time-adjusted analysis, participants drinking up to 0.5 L of wine per day (HR 0.83; 95%CI 0.74–0.92) and more than 0.5 L per day (HR 0.77; 95% CI 0.63–0.94) reported reduced mortality compared with non-drinkers (*p* = 0.0003) [152].

According to some authors in previous publications, the effect of ethanol on clotting factors and lipoproteins accounts for 50% of the beneficial effect of alcohol consumption in preventing atherosclerosis [121].

A number of studies assessing how various alcoholic beverages protect against CVD have found, as with red wine, a J-shaped relationship for increasing alcohol consumption and vascular risk [160].

The study team of Mendonça et al., assessed polyphenol intake in the Spanish population employing a validated semi-quantitative food frequency questionnaire.The collected information on food intake was then matched with the Phenol-Explorer database. The authors emphasized that although the questionnaire they used did not include some polyphenol-rich products, such as herbs and spices, there was no risk of misclassification, as the error would not be related to the occurrence or presence of the score. Based on the data collected, chocolate, coffee, cherries, apples, and olives were the main sources of variability in polyphenol intake. It was found that study participants with higher levels of flavonoid intake had a 47% lower incidence of cardiovascular events than those who consumed the least flavonoids (HR: 0.53, 95% CI: 0.29–0.98; P for trend Z 0.09). Notably, the results were not significant for other types of polyphenols [102].

Droste et al. [155] concluded that a moderate Mediterranean diet combined with exercise and an extra glass of red wine per day effectively independently improved the LDL/HDL ratio in patients with carotid atherosclerosis, even though the large majority were already on statin therapies, which suggests a synergistic effect of drugs as well as specific foods.

In assessing the relationship of consumption of alcoholic beverages with polyphenols, it should be considered that these compounds have different bioavailability. The most well-absorbed polyphenols in humans are isoflavones and gallic acid, followed by catechins [71], flavanones [161], and quercetin glucosides, with different kinetics [38,162]. The least well-absorbed polyphenols are proanthocyanidins, the galloylated catechins, and the anthocyanins [163]. There are no data for the other polyphenols available. Furthermore, gut microbiota probably metabolizes different polyphenols, which undoubtedly affects this class of compounds [164].

In conclusion, moderate consumption of wine positively impacts the progression and development of atherosclerosis due to its ethanol and polyphenols content confirmed by different biomarker levels. However, it is worth emphasizing that the health benefits may be intensified in women and people with high polyphenol absorption [121].

Panagiotakos et al. [148] in the Attica study observed a strong opposite and similar relationship of low wine or beer consumption when less than one glass per week was consumed, and risk of developing cardiovascular disease (HR: 0.40, 95% (CI): 0.17–0.98; and HR: 0.43, 95% CI: 0.20–0.93), respectively, in comparison with non-drinkers. The authors noted no significant relationship in individuals who had a consumption above one glass per week compared with abstainers. However, compared with subjects consuming less than two grams of ethanol per day, those consuming between 2 and 10, 10 and 20, and more than 20 g of ethanol per day had HRs (95% CI) of CVD risk of 0.60 (0.40–0.98), 1.22 (0.60–1.14), and 1.81 (0.70–4.61), respectively.

In contrast, da Luz et al. [147] reported the results of long-term follow-up of red wine (RW) drinkers in association with coronary calcification and clinical evolution. The researchers found that red wine users consumed an average of 28.9 ± 15 g of alcohol per day over 23.4 ± 12.3 years. Observation individuals had higher high-density and low-density lipoprotein levels but had lower C-reactive protein levels than abstainers. The risk of diabetes was lower among drinkers, but other risk factors were similar. It was also found that drinkers had higher Coronary Artery Calcification (CAC) index values than abstainers; the mean value was 131.5 ± 362 in drinkers vs. 40.5 ± 320 in abstainers (*p* <0.001). It was observed that major adverse cardiovascular events, MACE, was significantly lower in drinkers than in abstainers, despite their higher CAC. The authors concluded that this difference was mainly due to acute myocardial infarction (AMI) (0 vs. 6; *p* <0.03). Importantly, higher CAC values, in this case, did not predict a worse prognosis. The likely mechanism is lesion calcification, resulting in plaque stabilization and reduced clinical events [147].

Kechagias et al. [158] carried out a prospective randomized study in 2011. They indicated that red wine consumption in a moderate dose over three months increased hepatic triglyceride content (HTGC) in subjects free of hepatic steatosis at the baseline. However, as no participants developed hepatic steatosis, the authors suggest that the alcohol intake limit to defining non-alcoholic steatohepatitis should be no more than 150 mL for women and 300 mL for men.

In contrast, a prospective cohort study of Jani et al. [153] found that drinking red wine with food and staggered alcohol consumption over 3–4 days was connected with a reduced risk of fatality and vascular events for regular drinkers, after correcting for the influence of the mean alcohol intake. They said that drinking red wine was connected with a 48% lower risk of cirrhosis, 31% lower risk of MACE, 25% less chance of mortality, and 10% lower risk of alcohol-related accidents/self-harm than heavy alcohol.

Tresserra-Rimbau et al. [127] carried out an observational study within the PREDIMED trial to evaluate the association between intakes of total polyphenol and polyphenol subgroups and the risk of major cardiovascular events (myocardial infarction, stroke, or death from cardiovascular causes). They showed that hydroxybenzoic acids were strongly and inversely associated with CVD after controlling for potential confounders (HR Z 0.47; CI Z 0.26–0.86; *p*-trend: 0.02). However, it was observed that hydroxycinnamic acids (138–422 mg/d) were supplied with the highest amount from the diet, followed by hydroxybenzoic acids (6.9–36.1 mg/d) and the lowest amount of phenolic acids (0.1–17.9 mg/d).

Over the past five years, the topic of wine properties in research seems to have taken a different direction compared to an older period. Aside from the identification of compounds responsible for the cardioprotective properties of this beverage, research focusing on its action mechanisms, particularly resveratrol and other stilbenes, has become the leading trend.

In turn, a meta-analysis with 48,423 men and women showed that alcohol consumption with all assessed J-shaped outcomes compared to current non-drinkers, with a risk reduction that peaked at 7 g/day (relative risk 0, 79, 95% confidence interval 0.73–0.85) for all-cause mortality, 8 g/day (0.73, 0.64–0.83) for cardiovascular mortality, and 6 g/day (0.50, 0.26–0.96) for cardiovascular events, and remained significant up to 62, 50, and 15 g/day, respectively [17].

To summarize this section, although the French paradox has been studied for over 50 years, there are still areas that require well-designed, long-term randomized trials. As shown in the observational studies presented, alcohol correlates with better HDL outcomes but does not affect other parameters such as TG, LDL, lipoproteins vs. clotting factors, CRP, and oxidized LDL levels.

##### Clinical Trials

Earlier human studies demonstrated that short-term dietary intake interventions with ethanol in moderate amounts, red wine with ethanol, or sparkling wine low in polyphenols decreased the atherosclerosis risk as assessed by the appropriate biomarkers. Of note, the superior health status observed with the Mediterranean diet [6,18], which combines wine consumption in a mild range with a diet high in fruits, vegetables, and whole grains, implies that the wine polyphenols have a synergistic effect with the compounds present in other foods [165].

As mentioned before, there is a general consensus on a phenomenon of lower risk of cardiovascular disease in moderate drinkers, but scientists are still debating whether these effects are due to the presence of alcohol alone or to the non-alcoholic ingredients of wine such as polyphenolic compounds [132]. These issues may be solved only by performing randomized clinical trials assessing effects of wine compared to other alcoholic beverages. This is the only way to examine the diet of the participants in order to eliminate the antioxidative effect of other healthy compounds present in food such as vegetables and fruits. So far, the results of various clinical trials have proven contradictory. The results was shown in Table 6.

In contrast, data from the study by Di Renzo et al. [167] suggest that the combination of low/moderate intake of alcoholic beverages, with proven nutraceutical efficacy, and ethanol, combined with a Mediterranean diet may condition a reduction in atherosclerosis risk by positively modulating the expression of antioxidant genes that help prevent inflammatory and oxidative damage [167].

Taborsky’s IVIV study evaluated the effects of long-term regular red and white wine drinking on biomarkers of atherosclerosis. HDL level was assessed first, followed by total cholesterol, LDL, TG, LDLox, CRP, myeloperoxidase, Il-6, Il-18, matrix metalloproteinase, glutathione s-transferase, soluble CD40L, and fatty acid binding protein. According to several studies, white wine showed lower antioxidant capacity and antioxidant protective effects of LDL than red wine [159]. The investigators observed white wine. An inhibitory effect of red wine on the platelet-derived growth factor receptor (PDGFR) has also been demonstrated. Furthermore, a stronger anti-inflammatory effect was found for red wine compared to white wine consumption. However, wine drinking was shown to be associated with changes in lipid profile with unchanged liver function. The levels of gamma-glutamyl transferase, aspartate aminotransferase, and alanine aminotransferase did not change significantly [170]. Some data suggest that fermented, i.e., red, wine consumed in moderation results in the synthesis of conjugated linoleic acid (CLA), which has anti-atherosclerotic, anti-inflammatory, anti-adipogenic, hypotensive, and anti-diabetic effects. According to some scientific reports, the reduction in the risk of atherosclerosis is mainly due to the anti-inflammatory effect, which reduces the chronic inflammatory process occurring in large arteries such as the aorta and middle arteries, leading to the deposition of atherosclerotic plaques [171]. Results are also available confirming that polyphenols reduce macrophage oxidative stress by inhibiting NADPH oxidase, 15-lipoxygenase, cytochrome p450, and myeloperoxidase. Polyphenols from red wine are efficiently absorbed in humans and bind to LDL, protecting it from oxidation. The resveratrol in wine and other constituents also reduces the synthesis of lipids and eicosanoids that promote inflammation and atherosclerosis, protecting the heart from ischemia, reperfusion damage, and alleviating arteriosclerosis [164].

The hope is that in preventing secondary CVD, those who are presently drinkers may not have to stop drinking. However, it is important to inform them that the lower risk of death and following cardiovascular event is probably connected with reduced alcohol intake, i.e., up to a level of approximately 105 g (which is six and a half beer/cider pints, six and a half wine glasses or thirteen spirit shots) per week [17].

However, it must be understood that wine in any quantity is not recommended for pregnant women, infants, liver disease patients, and in conjunction with some medications. In addition, regular consumption of wine in people with a predisposition to alcoholism, cirrhosis of the liver, migraine headaches, and allergies should be taken with caution [58].

## 4. Food–Drug Interaction

Drugs do not only have interactions with each other but also with food components. Both chocolate and wine have documented cardioprotective properties, but the use of these products by cardiac patients, including those with atherosclerosis, is a separate issue. Those patients are on medication, very often on polytherapy, which increases the incidence of drug–food interactions. Various possible interactions may enhance, weaken, or completely change the effect of the drug. This narrative review summarizes the available data on interactions, especially of anti-atherosclerotic and lipolipemic drugs with phytochemicals present in chocolate and wine (polyphenols, flavon-3-ols, and stanols) and alcohol. The physician should inform the patient of possibility of alternation of the therapy effectiveness by nutrients provided with the diet. Drug–food interactions can occur both in the pharmacodynamic phase, where synergism and antagonism occur, and in all stages of the pharmacokinetic phase (LADME). Moreover, chronic alcohol abuse leads to stimulation of the microsomal enzyme system, increasing drug metabolism and elimination. This area is important because it is possible to use additive effects of statins and diet and harness existing synergies in order to lower the intensity of the drug or dose. As for pharmacotherapy, the latest recommendations of the European Society of Cardiology (ESC) and the European Atherosclerosis Society (EAS), include only three drugs for the management of dyslipidemias: statins, ezetimibe, and a PCSK9 inhibitor, but others are still in use, the characteristics of which are given in Table 7 [116].

The recommendations cover four situations:

(1) a statin as the drug of choice to improve prognosis in individuals with TG >200 mg/dL;
(2) patients with TG 135–499 mg/dL—irrespective of statins, administration of omega-3 fatty acids in a dose of 2 × 2 g/day is recommended;
(3) in primary prevention, reasonable LDL-cholesterol control TG >200 mg/dL fenofibrate or benzafibrate, together with statins;
(4) high cardiovascular risk groups, with reasonable LDL-cholesterol control with TG > 200 mg/dL, fenofibrate or benzafibrate together with a statin [116].

Although drug–food interactions are considered crucial for the efficacy of treatment in various diseases, the mechanisms of many interactions of CVD and anti-atherosclerotic drugs with chocolate and wine have not yet been studied. Little is currently known about the interaction of wine with statins and antiplatelet drugs [1,38,39].

However, interactions may occur with different components of chocolate and wine, especially polyphenols, and within this group of compounds, drug interactions with resveratrol, followed by quercetin, have been described most extensively. The results of studies on interactions between aspirin and gallic acid found in wine are also available [38]. It should be emphasized that the pharmacokinetics and pharmacodynamics of drugs can be altered by the presence of macronutrients such as fat or carbohydrate [42]. Thus, lipophilic drugs are better absorbed in the presence of fat; thus, simvastatin, atorvastatin, and lovastatin are likely to be better absorbed after consuming chocolate, whereas fluvastatin and pravastatin, due to their hydrophilic nature, in the presence of wine [43]. Another point to note is the effect of the pH of the food. Wine with a pH of 3.5–4.0 causes acidification of the gastrointestinal contents, reduces the absorption of alkaline drugs, and increases the absorption of acidic drugs [44].

The effect of food on various drugs is difficult to determine conclusively. Although the data are summarized, they are often contradictory and disparate; therefore, their scope is limited. In some cases, there is likely to be unrecognized synergism. For instance, virtually no data are currently available describing interactions of statins with components of the diet or dietary patterns in subgroups of the population, especially those most likely to benefit if positive effects are found. Therefore, it is practically not possible at this time to reach any firm conclusions [173].

### 4.1. Chocolate–Drug Interaction

The best-known interactions of chocolate, which contains small amounts of tyramine, with drugs that are MAO inhibitors are described in the available literature [82].

Additionally, interactions of chocolate have been documented with drugs such as antibiotics—e.g., ciprofloxacin or some bronchodilators such as theophylline [174].

Mergenhagen et al., reported a case of heart block following the concomitant administration of linezolid with tyramine at a dose of 7 mg [175]. According to the recommendations, beverages and products containing a high content of tyramine should be avoided, especially when administering drugs from the group of monoamine oxidase inhibitors. The sympathetic response was compared by recording blood pressure in patients receiving linezolid and tyramine with the control group receiving linezolid with placebo. In the study group, an average increase in systolic blood pressure by 30 mm Hg was observed [176].

In contrast, in a study of 30 patients in double-blind crossover studies with 200 mg of tedizolid and oral tyramine challenge, both the control and study groups showed an increase in mean arterial pressure of 30 mmHg with 325 mg of tyramine. When using this drug, there are no restrictions on the diet rich in tyramine [176].

In a double-blind study, 37 type 2 diabetic patients on statin treatment were randomly assigned to consume a chocolate containing 2.1 g of plant sterol esters or receive ezetimibe 10.0 mg/day for 6 weeks. It was observed that ezetimibe promoted an increase of superoxide dismutase and glutathione reductase activities, while plant sterols did not change these biomarkers. Among individuals who showed the greatest LDL-C and oxLDL-C reduction, the oxLDL-C reduction observed in individuals under plant sterol treatment (−20.0%) did not differ from the values found in individuals treated with ezetimibe (−21.1%). In addition, 16 patients on plant sterol treatment reached the optimal therapeutic goal for LDL-C (<70 mg/dL) [115].

Curtis et al. [64], on the other hand, conducted a parallel-design, placebo-controlled study involving female patients with type 2 diabetes consuming 27 g/day (divided dose) of flavonoid-enriched chocolate (containing 850 mg flavan-3-ols (90 mg epicatechin) and 100 mg isoflavones or matched placebo per year. The authors found that compared with the placebo group, the combined flavonoid intervention resulted in significant reductions in total cholesterol: HDL-cholesterol (HDL-C) (−0.2 ± 0.1; *p* = 0.01) and LDL-cholesterol (LDL-C) (−0.1 ± 0.1 mmol/L; *p* = 0.04) ratios. The estimated 10-year total risk of coronary heart disease was reduced after the flavonoid intervention (flavonoid +0.1 ± 0.3 vs. placebo 1.1 ± 0.3; *p* = 0.02). Moreover, there is an additional benefit of flavonoids over standard drug therapy in managing CVD risk in postmenopausal patients with diabetes mellitus type 2 [64].

Statin intolerance, real or perceived, is an increasingly common problem in clinical practice. The study by Scolaro et al. [176] aimed to evaluate the effects of statin therapy at a reduced dose supplemented with nutraceuticals. Patients (n ¼ 10) were selected and recruited for a pilot statin dose reduction protocol based on multivariate analysis. Patients were given chocolate containing plant sterols at 2.2 g/day and green tea (two sachets/day) for 6 weeks. Initially, 53 patients with type 2 diabetes treated with statins received fish oil supplementation at a dose of 1.7 g EPA and DHA/day. Supplementation was conducted for 12 weeks: standard statin therapy was maintained for the first 6 weeks and reduced by 50% from week 6 onwards. After six weeks of supplementation, plasma LDL-C decreased by 13.7% ± 3.7, P¼ 0.002 and C-reactive protein by 35.5% ± 5.9, as did the rate of cholesterol absorption but not cholesterol synthesis. No difference was observed for plasma lipids, inflammation, and cholesterol efflux P ¼ 0.03) in the study’s second phase with a reduced dose of statins. Analysis of plasma lathosterol and campesterol suggested that the intensity of LDL-C reduction was mediated by capacity or HDL particles after reduction in statin dose compared with standard therapy. Despite the study’s limitations related to the small sample size, the researchers highlight the potential of a new therapeutic approach combining a reduced statin dose and specific compounds in the diet. These results may benefit many patients with CVD or at risk of CVD who cannot tolerate high-dose statin therapy [177].

Undoubtedly, a limitation of these studies is the use of chocolate enriched in flavonoids, which is not practical in everyday life, so the results of these studies need solid long-term clinical trials.

It should also be added that the interaction of chocolate with statins may occur due to the presence of phenolic compounds whose hepatic metabolism, like that of selected satins, usually follows the cytochrome CYP3A4 isoform.

Possible interactions for which the results are unknown include taking statins with chocolate, which as a high fat product may increase the absorption of lipophilic drugs such as atorvastatin, simvastatin, ezetimibe, and others and inhibit the absorption of hydrophilic drugs. However, in the case of antiplatelet drugs, some studies are available suggesting that chocolate acts synergistically with them and this may lead to bleeding as a result of COX-I inhibition [41].

### 4.2. Wine–Drug Interaction

Red wine is a crucial component of the Mediterranean diet, but its consumption is not allowed in most pharmacotherapy regimens [6,18,142]. Information on interactions with alcohol is widely available both in the available scientific literature and in online pharmaceutical encyclopedias such as drugs.com or Drug Bank.

Although drug administration and alcohol consumption during pharmacotherapy are controversial, some researchers found a beneficial effect of moderate alcohol on lipid levels in patients treated with statins. Zdrenghea et al., in a clinical trial showed that concomitant administration of the simvastatin and consumption of 20 g of alcohol per day increased HDL-C values and reduced serum LDL-C levels by 40%, compared with the administration of drug alone [178]. However, the study had a serious limitation of having a small group of 20 subjects [178].

In contrast, Smith et al. [179] evaluated the effect of moderate alcohol drinking (20 g/day) on the efficacy and safety of fluvastatin therapy. The results showed a moderate effect of alcohol on the pharmacokinetic parameters of fluvastatin; this was a slight increase in AUC and a decrease in C_max_. Alcohol administration at a dose of 20 g/day did not worsen the hypolipemic properties of the drug. Authors also assessed the effect of a higher dose of alcohol (70 g/day) on the pharmacokinetics of fluvastatin and observed a slight decrease in the AUC of the study drug. The administration of fluvastatin with 70 g/day of alcohol did not change the LDL-C-lowering effect in the serum of the subjects, but increased triglyceride levels compared with taking the drug alone. However, they observed large variability in the results in pharmacokinetic parameters obtained in individual subjects. The authors point to the need for further studies. No publications were found on the concomitant administration of atorvastatin and alcohol in humans. Therefore, consumption of small amounts of dietary alcohol (20 g/day) used together with simvastatin or fluvastatin did not attenuate their hypolipemic effect; and even in the case of simvastatin, this amount of alcohol had a beneficial effect manifested by an increase in HDL-cholesterol levels [178]. On the other hand, the combined use of statins (e.g., simvastatin, atorvastatin) with alcohol leads to an inhibition of CYP450 3A4-mediated metabolism of these drugs and an increase in their serum concentration as a result of share hepatic metabolism [39]. It is worth noting that in larger quantities, chronic alcohol consumption is generally contraindicated during statin therapy as it may increase the risk of liver damage and rhabdomyolysis [178].

Interference with the bioavailability of statins due to competition of flavonoids with CYP450 enzymes, esterases, uridine diphosphate glucuronosyltransferases, and transporters (P-glycoprotein, multidrug resistance proteins, organic anion transporting polypeptides, monocarboxylic transporters) should also be considered. Due to the low substrate specificity of the transporters, disturbances in the bioavailability of statins and any drugs transported with the above-mentioned carriers should be considered. Long-term supply of red wine rich in flavonoids may modulate the expression of transporter genes and enzymes, and therefore it may lead to an increase in the clearance of statins. In the future, particular attention should be paid to the problem of the interaction of statins with red wine and studies of the interaction between prescribed statin and food should be carried out [180].

When acetylsalicylic acid is taken together with wine, there is a synergistic interaction and enhancement of the antiplatelet effect due to inhibition of cyclooxygenase I (COX-I) in platelets, with consequent bleeding [40,41]. However, larger-scale studies are now being conducted to determine the effects of polyphenols and their main representative resveratrol on drug pharmacokinetics and pharmacodynamics [39,181]. Many studies have investigated the effect of red wine components on CYP3A4 activity. However, some research has shown that trans-resveratrol may non-competitively inhibit or inactivate CYP3A4 and CYP3A5 and modulate CYP activity at the level of gene transcription [182]. Hyrsova et al. [181] demonstrated, that natural resveratrol derivatives (trans- and cis-RSV, oxyresveratrol, pinostilbene, and pterostilbene) significantly inhibit CYP3A4/5 enzymatic activities; however, only trans-RSV significantly inhibits CYP3A4/5 activity (both testosterone 6β-hydroxylation and midazolam 1′-hydroxylation) in micromolar concentrations by a non-competitive mechanism, suggesting a potential risk of food–drug interactions with CYP3A4/5 substrates [181].

It is of note, however, that resveratrol is most probably not one of the principal components of red wine that induces CYP3A4 inactivation. In fact, it was reported that red wine fractions without resveratrol also significantly inhibited CYP3A4 in vitro. Furthermore, the resveratrol found in red wine was too low to account for the degree of CYP3A4 inactivation observed after red wine treatment. These conclusions were confirmed by inactivation studies using different types of red wine. This fact demonstrated that the level of resveratrol did not correlate with CYP3A4 inactivation, as found in a previous study by Chan and Delucchi [183].

Due to the high risk of cardiometabolic drug–food interactions, which are dangerous to the health and life of patients, solid clinical studies to ensure the safety and efficacy of pharmacotherapy must be carried out in the near future. However, preclinical interaction does not always refer to a clinical practice, which influences the optimization of pharmacotherapy by health professionals [184].

## 5. Conclusions

Although research on the cardioprotective properties of the products discussed in the review has been conducted for years, knowledge is still incomplete at present and further extensive studies need to be undertaken. The review confirmed the cardioprotective properties of investigated products when consumed in moderation. However, it should be emphasized that the high energy value of these products, the complex composition of the matrix, and the lack of guidance on the amount of consumption may lead to adverse effects. As is well known, excessive alcohol consumption is a major cause of premature death and excess calories lead to obesity and other related non-communicable diseases. On the other hand, there is little research on the interactions of polyphenols with other food components and drugs. It seems advisable to carry out extensive research in this direction. The adverse effects of excessive alcohol and caloric intake have been comprehensively described in the literature. In contrast, there are few data on the interaction of polyphenols with drugs taken in patients with cardiovascular disease.

## Figures and Tables

**Table 1:** Chemical composition of chocolate and wine [78].

Chemical Compounds	Food Products	
	Dark Chocolate	Red Wine
Water (g)	0.6	89.9
Protein (g)	6.7	0.1
Lipid (g)	34.3	0
Cholesterol (mg)	0	0
Carbohydrate (g)	56.6	0.2
Sugar (g)	38.3	0.2
Total fiber (g)	1.7	1.7
Sodium (µg)	4000	7000
Potassium (µg)	581,000	110,000
Iron (µg)	21,000	1000
Calcium (µg)	42,000	7000
Phosphorum (µg)	244,000	13,000
Thiamin (µg)	40	0
Riboflavin (µg)	10	10
Niacin (µg)	46	46
Vitamin A (µg)	0	0
Energy (kcal/kJ)	593/2330	68/284

**Table 2:** Polyphenolic compounds in chocolate and wine.

Parameter	Chocolate		Wine	
	[mg/kg]		[mg/L]	
Total polyphenols ng/GAE/L	11.7–14.8		860.2–2912.0	
Dihydroflavonols	-		Dihydromyricetin 3-O-rhamnoside 45.0	
Flavanols	(+)-Catechin (−)-Epicatechin (−)-Epicatechin-(2a-7)(4a-8)- picatechin 3-O-galactoside Cinnamtannin A2 Procyanidin dimer B2 Procyanidin trimer C1	107.0–500.0 32.74–125.0 40.0–80.0 290.0–860.0 210.0–540.0 130.0–440.0	(+)-Catechin (+)-Gallocatechin (−) Epicatechin (−)-Epicatechin 3-O-gallate (−)-Epigallocatechin	14.0–390.0 0–4.0 0–165.0 0–9.0 6.0
Flavanones	-		Hesperetin Naringenin Naringin	0.5–0.6 0.4–0.7 7.0–8.0
Flavonols	Quercetin	250.0	Kaempferol Kaempferol 3-O-glucoside Myricetin Quercetin Quercetin 3-O-arabinoside Quercetin 3-O-glucoside Quercetin 3-O-rhamnoside Quercetin 3-O-rutinoside	0–3.6 6–11.0 0–18.0 0–32.0 4.0–5.0 8.0–23.0 0–18.0 0–32.0
Hydroxybenzoic acids	-		Gallic acid Gallic acid ethyl ester	0–126.0 14.0–17.0
Hydroxycinnamic acids	Ferulic acid	240.0	2,5-di-S-Glutathionyl caftaric acid Caffeoyl tartaric acid Caffeic acid Ferulic acid	11.0–47.0 0 0–77.0 0–10.0
Stilbenes	Resveratrol Resveratrol 3-O-glucoside	0.4 1.0	Resveratrol Resveratrol 3-O-glucoside	0.4 75.0–964.0
Tyrosols	-		Tyrosol Hydroxytyrosol	5.0 6.0
Total anthocyanogens	2.8–4.1		21.0–1011.0	
Antioxidant activity mmol TE/L	151.7–246.0		6.9–15.2	
Main group	flavan-3-ol		stilbenes	

Mean value of reference data from Phenol-Explorer Database (version 3.6, Paris, France) [86].

**Table 3:** Summary of studies assessing the effects of cocoa/dark chocolate on the cardiovascular system.

Investigation	Population	Outcomes	Ref.
FQQ	men & women 32,486/40,009	I. AF 9978 participants II. CR vs. NCR↑AF ≥34 srv/week 0.96 (95% CI 0.88–1.04) III. HF AF: 0.97 (95% CI 0.94–1.01): 2 srv/week ↑ DCC 0.96 (95% CI 0.90–1.03)—HC vs. LC	[92]
FFQ	55,502	I. 1–3 srv/month (HR 0.90, 95% CI 0.82 to 0.98) II. 1 srv/week (HR 0.83, 95% CI 0.74 to 0.92) III. 2–6 srv/week (HR 0.80, 95% CI 0.71 to 0.91) IV. ≥1 srv/day (HR 0.84, 95% CI 0.65 to 1.09) men ↔ women	[91]
FFQ	men & women 2,157/31,917	I. 1–3 srv/month (HR 0.88, 95% CI 0.78 to 0.99) II. 1–2 srv/week (HR 0.83, 95% CI 0.72 to 0.94) III. 3–6 srv/week (HR 0.82, 95% CI 0.68 to 0.99) IV. ≥1 srv/day (HR 1.10, 95% CI 0.84 to 1.45)	[90]
HEI-2005	15,023 adults	↑HDL-c ↓TG ↓CRP levels, ↓weight	[94]
FFQ	men & women 38,182/46,415	DCC↓risk of stroke (women)	[87]
I: 50 g DC II.50 g/mc wash-out period (7 days)	21	single dose 50 g DCC ↔ EFP & MFP	[95]
FFQ	590	↓ DCC↑risk DM2	[96]

AF—atrial fibrillation, CI—confidence interval; CR—consumer; CRP—C-reactive protein; CVD—cardiovascular disease; DC—dark chocolate; DCC, dark chocolate consumption; DM2—Diabetes mellitus type 2; EFP—endothelial function in patients; FFQ—food frequency questionnaire; DC—highest consumption; HDL-c—high-density lipoprotein; HEI-2005—Healthy Eating Index (2005); HR—hazard ratio; LC—lowest consumption; MFP—microvascular function in patients; NCR—non-consumer; TG—triglycerides; srv—serving; Explanation of arrows: ↔ no change; ↑ improvement/increase; ↓ decrease.

**Table 4:** Summary of major RCT studies evaluating chocolate intake and cardiovascular outcomes.

Intervention	Population	Findings	Ref.
CG: 20 g DC (500 mg polyphenols) CC: 20 g placebo DC	42	↔ lipid profile ↓ SBP, DBP ↔ body weight, BMI	[106]
20 mL drink 500 mg theobromine	44	I. THB ↔ ABCA1-MC_ef_ II. THB↑miR-92a levels III. HFM e ↑PC_ef_ and 3 selected miRNAs levels	[65]
50 g DC	65	↑mRNA expression of the AIC IL-10 ↓ intracellular pro-inflammatory stress response AI_ef_	[107]
I. 70 g DC (150 mg ECT) + placebo capsules II. pure ECT capsules (2 × 50 mg ECT) + 75 g MW III. placebo capsules + 75 g MC (0 ECT)	20	↑VF after pure ECT ↔ DC	[108]
DC (>85% cocoa)	24	supplements of DC↑redox state ↓ biomarkers of muscle damage	[109]
85% cocoa DC 1 g/kg or MC	47	I. DC↑SBP and DP at rest but buffered the reactivity of DBP, HR, MAP, & DP during MS II. DC buffer cardiovascular reactivity	[72]
I. (AD) II. 42.5 g/day ALD III. 18 g/day CPr & 43 g/day of DC IV. all 3 foods (DC + ALD).	48	ALD alone or mix DC under controlled-feeding conditions ↓ lipid profiles	[110]
60 g DC + flavan-3-ols & procyanidins (standard DC and MC)	42	flavan-3-ol-enriched DC & only DC: I. ↓ UC, L, AA II.↑pyruvate & 4-hydroxyphenylacetate, phenolic compound of bacterial origin.	[65]
20 g LCC »55% cocoa; 20 g HCC; »90% cocoa	30	consumption of HCC: ↑VF; ↓ reducing CBAP ↑VR,	[111]
50 g DC & 30 g CPr	136	7-d cocoa-based supplementation↑conditions PL	[112]
40 g DC (cocoa >85%) vs. LC MC (cocoa <35%) polyphenols	57	CPC ↑ EF via Nox2 down-regulation in NASH patients.	[113]

AI_ef_—anti-inflammatory effects; DC—dark chocolate; MC—milk chocolate; FMD—flow-mediated vasodilatation; NASH—non-alcoholic steatohepatitis; Nox2—oxidase isoform; CPC—cocoa polyphenols consumption; CPr—cocoa powder, LCC—lower cocoa chocolate; HCC—high cocoa chocolate, ALD—almond diet; CHOC—chocolate diet; ABCA1—cholesterol-efflux transporter ABCA1; MAP—mean arterial pressure; ECT—epicatechin; AD—American diet; HFM—high fat meal; EF—endothelial function; LC—low content; VF—vascular function; CBAP—central brachial artery pressures; vascular relaxation—vascular relaxation; UC—urinary creatinine; L—lactate; AA—amino acids; PL—plasma lipids; PC_ef_—postprandial cholesterol efflux; MC_ef_ —mediated cholesterol efflux; AIC—anti-inflammatory cytokine; DBP—diastolic blood pressure, SBP—systolic blood pressure; MS—mental stress, Explanation of arrows: ↔ no change; ↑ improvement/increase; ↓ decrease.

**Table 5:** Summary of studies assessing evaluating relationship wine and alcohol beverages on CVD.

Study Designed	Population	Findings	Ref.
MORGAM Project	142,960	I. AI <10 g/day ~ 11%↓RTM, II. AI >20 g/day—13%↑RTM, III. AI >20 g/day—22%↑RTM, IV. AI <1 drink/day ↓ RTM and CVD, V. AI >2 drinks/d↑RTM	[156]
Questionnaires. Phenol-Explorer database	17,065	↑intake flavonoids ~ 47%↓incidence of CVD	[102]
RW 100 mL (women) or 200 mL (men) daily (20 weeks)	108	Lch↓LDL/HDL ~ 8% (*p* = 0.0242) and RW ~13% (*p* = 0.0049) TCh↓6%; (*p* = 0.0238) and TC↓13%; (*p* = 0.0361) Lp (a) ↔ any intervention.	[155]
Hydroxytyrosol and HVAL in urine samples	1851	I. all biomarkers↓CVD risk, II. HVAL strong inverse association, III. HVAL RTM	[157]
1 glass of RW 4–5 day/week (5 years) 28.9 ± 15 g of EtOH/d for 23.4 ± 12.3 years.	354	I. drinkers > CAC abstainers, II. drinkers vs. abstainers↑HDL & LDL, ↓CRP III. drinkers vs. abstainers MACE was significantly↓, despite higher CAC.	[147]
150 mL of RW/day women & 300 mL RW (33 g EtOH/d) men	46	I.↑HTGC (during 3 months), II. no subject developed hepatic steatosis, III. LDL-ch↓by RW	[158]
Questionnaires.: I. abstainers and occasional consumers, II. beer consumers, III. consumers of mixed drinks	240	MAI vs. abstainers -↑HDL-c & adiponectin,	[142]
WI never/ WI almost never, WI<0.5 L/day, WI >0.5 L/day	11,248	I. MWI vs. abstainers at baseline↓risk of CVD II. MWI↓RTM	[152]
I. no use II. ≤1 glass/week III. >1 glass/week	2583	I. wine/beer (≤1 glass/week)↓vs. abstention, II. <2 g/day EtOH, 2–10, 10–20, >20 g/d—CVD-risk HRs	[148]
150 mL MW, WW or RW (2 years). MedDiet	224	RW↑HDL, HDL-C & apolipoprotein(a)1 ↓the total cholesterol–HDL-C	[154]
Effects of RW and WW on atherosclerosis	157	RW and WW ↔ clinically relevant differences in lipid profile, CRP, blood glucose and other markers of atherosclerosis	[159]
FFQ Phenol-Explorer database	273	I. 46%↓in risk of CVD of TP intake II. the polyphenols = strongest inverse associations: flavanols, lignans and hydroxybenzoic acids	[127]
Questionnaires	502,616	↑RTM compared to participants drinking EtOH↑3–4 days in a week	[153]

AI—alcohol intake; CAC—coronary artery calcification, CVD—cardiovascular disease; EtOH—ethanol, FFQ—food frequency questionnaire, HDL—high-density lipoprotein cholesterol; HVAL—homovanillyl alcohol; LDL—low-density lipoprotein cholesterol; MACE—major adverse cardiac event; MAI—moderate alcohol intake; MWI—moderate wine intake; RTM—risk of total mortality; RW—red wine; TP—total phenols; WI—wine intake; WW—white wine; Explanation of arrows: ↔ no change; ↑ improvement/increase; ↓ decrease.

**Table 6:** Summary of major RCT studies evaluating wine intake and cardiovascular outcomes.

Intervention	No. of Pts.	Findings	Ref.
90 day treatment with RSV (300 mg, 1000 mg and placebo)	65	sVCAM-1 and tPAI↑ >1000 mg vs. 300 mg vs. placebo groups	[139]
RSV—125 mg/day or 500 mg/day or placebo	66	RSV ↔ walking performance in people with PAD age >65	[140]
WW (2 drinks/day), WW + TYR (25 mg) and water (control) ad libitum.	33	TYR and OHTYR ↑CVD	[54]
I. no EtOH, II. 70 mL of tsipouro 38% ABV III. 200 mL RW 13.5% ABV. consumed of EtOH—27 g/day, (8 weeks)	57	8 weeks RWI ↓BCS from peripheral blood cells	[141]
RW (150 + 100 mL of water), IPA (250 mL), blonde (250 mL), and free beer (250 mL).	20	TYR absorption & endogenous conversion HT	[132]
AAW or gin (0.5 g EtOH/kg)	41	↑TLR4, TLR6 and Caspase-1 ↓TLR2, Interleukin-1 receptor, chemokine receptor 3 & inflammasome expression↓chemokine receptor 5↓	[53]
RW or VDK (3 units/day)	77	↑levels of leptin (after RW and VDK), ↑levels of APO A1 (after VDK), ↑adiponectin	[166]
(a) fasting +30 g of EtOH from RW, (b) fasting +30 g of EtOH from WW, (c) fasting +30 g of EtOH from VDK, (d) MeDM, (e) HFM, (f) MeDM +30 g of ethanol from RW, (g) MeDM +30 g of ethanol from WW, (h) MeDM +30 g of ethanol from VDK, (i) HFM +30 g of ethanol from RW, (j) HFM +30 g of ethanol from WW, and (k) HFM +30 g of ethanol from VDK	55	MAI + nutraceuticals + EtOH + MeDM ↓the risk of atherosclerosis	[167]
150 mL RW, WW or water	224	↓Apo(B)/Apo(A) (no progression in carotid-TPV)	[168]
200 mL/day RW (4 weeks)	24	RW ↔ IL-6, TNF-α and CRP overweight subjects ↑adiponectin ↑antioxidant potential of LDL ↑ in paraoxonase & adiponectin	[145]
230 mL/d (∼24g alcohol/d) RW—women, 300 mL/day (∼31g alcohol/d) RW—men or Eq volumes of DRW or water (4 weeks)	24	RW↑BP by 2.5 ± 1.2/1.9 ± 0.7mmHg, RW ↔ glycemic or other CRF	[169]
gin (100 mL/day, 30 g EtOH), RW (272 mL/day, 30 g EtOH and 798 mg TP), or DRW (272 mL/day, 1.14 g EtOH & 733 mg TP) (4 weeks)	67	glucose ↔, plasma insulin & HOMA-IR↓(RW & DRW), HDL, apolipoprotein A-I & A-II ↑ (RW & gin), Lipoprotein↓(RW)	[58]

AAW—Andalusian aged wine; ABV—Alcohol By Volume; BP—blood pressure; BCS—basal cytokine secretion; CRF—cardiovascular risk factor; DRW—dealcoholized red wine; EtOH—ethanol; HFM—high-fat meal; IPA—Indian pale ale beer; MeDM—Mediterranean meal; MAI—modrate alcolol intake; OHTYR—phenol hydroxytyrosol; RSV—resveratrol; RW—red wine; sVCAM-1—soluble vascular cell adhesion molecule-1; TLR4—Toll-like receptors 4; TLR6—Toll-like receptors 6; tPAI—total plasminogen activator inhibitor; TP—total polyphenols; TPV—total plaque volume; TYR—tyrosol; VDK—vodka; WW—white wine; Explanation of arrows: ↔ no change; ↑ improvement/increase; ↓ decrease.

**Table 7:** Characteristic of anti- atherosclerotic and hypolipemic drugs.

Group of Drug	Name of Drug	Mechanism	Solubility	Liver Metabolism
Statins	Atorvastatin Rozuvastatin Fluvastatin Lovastatin Simvastatin Pitavastatin Pravastatin	competitively inhibits 3-hydroxy-3-methylglutaryl-coenzymeA (HMG-CoA) reductase,	Lipophilic Hydrophilic Lipophilic Lipophilic Lipophilic Lipophilic Hydrophilic	CYP450 3A4 CYP450 2C9 and 2C19 CYP450 2C9 (minor) CYP450 3A4 CYP450 3A4 CYP450 2C9 (minor), glucuronidation
Fibrate	Fenofibrate Ciprofibrate	Fenofibrate activates peroxisome proliferator activated PPARα, increasing lipolysis, activating lipoprotein lipase, and reducing apoprotein C-III	Lipophilic	weak inhibitors of CYP2C19 and CYP2A6, weak do moderate inhibitors of CYP2C9.
Inhibitor PCSK9	Evolocumab Alirocumab	PCSK9 inhibitors, negative regulation of DLR		Do not induc enzyme
Cholesterol absorption inhibitors	Ezetimibe	Associates with the protein responsible for steroid uptake into the cell—Niemann-Pick C1 like 1 (NPC1L1) in the intestinal mucosal epithelium.	Lipophilic	Does not induce enzyme
Antiplatelet drugs	Ticlopidyna Clopidogrel ASA	inhibit platelet activation and aggregation by irreversibly blocking the ADP P2Y12 receptor. inhibition of the enzyme COX-1, inhibiting prostaglandin production, stopping the conversion of AA to TXA2	Hydrophilic	CYP1A2, CYP2B6, CYP2C9, CYP2C19, and CYP3A4/5
Direct oral anticoagulants (DOACs) offers	Rivaroxaban Dabigatran Apixaban Edoxaban	Competitively inhibit free and clot bound factor Xa.	Hydrophilic	Although, in theory, food or herbal inhibitors/inducers of CYP3A4 or P-gp might interfere with the pharmacokinetics of DOACs, no direct evidence of such interactions exist
PUFA	n-3: EPA’ DHA	EPA i DHA precursors of prostaglandins, thromboxanes and leukotrienes	Lipophilic	(CYP7A1) [172]

## Data Availability

Not applicable.

## References

[1] Borén J., John Chapman M., Krauss R.M., Packard C.J., Bentzon J.F., Binder C.J., Daemen M.J., Demer L.L., Hegele R.A., Nicholls S.J. (2020). Low-density lipoproteins cause atherosclerotic cardiovascular disease: Pathophysiological, genetic, and therapeutic insights: A consensus statement from the European Atherosclerosis Society Consensus Panel. Eur. Heart J..

[2] Zhang Y., Koradia A., Kamato D., Popat A., Little P.J., Ta H.T. (2019). Treatment of atherosclerotic plaque: Perspectives on theranostics. J. Pharm. Pharmacol..

[3] Teissedre P.L., Stockley C., Boban M., Gambert P., Alba M.O., Flesh M., Ruf J.C. (2018). The effects of wine consumption on cardiovascular disease and associated risk factors: A narrative review. Oeno One.

[4] Bittner V. (2020). The New 2019 AHA/ACC Guideline on the Primary Prevention of Cardiovascular Disease. Circulation.

[5] Yokose C., McCormick N., Rai S.K., Lu N., Curhan G., Schwarzfuchs D., Shai I., Choi H.K. (2020). Effects of low-fat, mediterranean, or low-carbohydrate weight loss diets on serum urate and cardiometabolic risk factors: A secondary analysis of the dietary intervention randomized controlled trial (direct). Diabetes Care.

[6] Morales G., Martínez-González M.A., Barbería-Latasa M., Bes-Rastrollo M., Gea A. (2021). Mediterranean diet, alcohol-drinking pattern and their combined effect on all-cause mortality: The Seguimiento Universidad de Navarra (SUN) cohort. Eur. J. Nutr..

[7] Temple N.J., Guercio V., Tavani A. (2019). The Mediterranean Diet and Cardiovascular Disease: Gaps in the Evidence and Research Challenges. Cardiol. Rev..

[8] Schwingshackl L., Morze J., Hoffmann G. (2020). Mediterranean diet and health status: Active ingredients and pharmacological mechanisms. Br. J. Pharmacol..

[9] Sayón-Orea C., Razquin C., Bulló M., Corella D., Fitó M., Romaguera D., Vioque J., Alonso-Gómez Á.M., Wärnberg J., Martínez J.A. (2019). Effect of a Nutritional and Behavioral Intervention on Energy-Reduced Mediterranean Diet Adherence Among Patients With Metabolic Syndrome Interim Analysis of the PREDIMED-Plus Randomized Clinical Trial. JAMA.

[10] Martinotti S., Bonsignore G., Patrone M., Ranzato E. (2021). Mediterranean Diet Polyphenols: Anthocyanins and their Implications for Health. Mini-Rev. Med. Chem..

[11] Bazal P., Gea A., Martínez-González M.A., Salas-Salvadó J., Asensio E.M., Muñoz-Bravo C., Fiol M., Muñoz M.A., Lapetra J., Serra-Majem L.L. (2019). Mediterranean alcohol-drinking pattern, low to moderate alcohol intake and risk of atrial fibrillation in the PREDIMED study. Nutr. Metab. Cardiovasc. Dis..

[12] Esposito K., Maiorino M.I., Bellastella G., Panagiotakos D.B., Giugliano D. (2017). Mediterranean diet for type 2 diabetes: Cardiometabolic benefits. Endocrine.

[13] Drehmer M., Odegaard A.O., Schmidt M.I., Duncan B.B., de Oliveira Cardoso L., Matos S.M., Maria del Carmen B.M., Barreto S.M., Pereira M.A. (2017). Brazilian dietary patterns and the dietary approaches to stop hypertension (DASH) diet-relationship with metabolic syndrome and newly diagnosed diabetes in the ELSA-Brasil study. Diabetol. Metab. Syndr..

[14] Acosta-Navarro J.C., Oki A.M., Antoniazzi L., Bonfim M.A.C., Hong V., De Almeida Gaspar M.C., Sandrim V.C., Nogueira A., Aparicio H.J., Benjamin E.J. (2019). Consumption of animal-based and processed food associated with cardiovascular risk factors and subclinical atherosclerosis biomarkers in men. Rev. Assoc. Med. Bras..

[15] Visioli F., Panaite S.A., Tomé-Carneiro J. (2020). Wine’s phenolic compounds and health: A pythagorean view. Molecules.

[16] Zatońska K., Psikus P., Basiak-Rasała A., Stępnicka Z., Wołyniec M., Wojtyła A., Szuba A., Połtyn-Zaradna K. (2021). Patterns of alcohol consumption in the pure poland cohort study and their relationship with health problems. Int. J. Environ. Res. Public Health.

[17] Ding C., O’Neill D., Bell S., Stamatakis E., Britton A. (2021). Association of alcohol consumption with morbidity and mortality in patients with cardiovascular disease: Original data and meta-analysis of 48,423 men and women. BMC Med..

[18] Minzer S., Estruch R., Casas R. (2020). Wine Intake in the Framework of a Mediterranean Diet and Chronic Non-Communicable Diseases: A Short Literature Review of the Last 5 Years. Molecules.

[19] Ho Y.-L., Nguyen X.-M.T., Yan J.Q., Vassy J.L., Gagnon D.R., Gaziano J.M., Wilson P.W., Cho K., Djoussé L. (2021). Chocolate consumption and risk of coronary artery disease: The Million Veteran Program on behalf of the VA Million Veteran Program. Am. J. Clin. Nutr..

[20] Garcia J.P., Santana A., Baruqui D.L., Suraci N. (2018). The cardiovascular effects of chocolate. Rev. Cardiovasc. Med..

[21] Gammone M.A., Efthymakis K., Pluchinotta F.R., Bergante S., Tettamanti G., Riccioni G., Orazio N.D. (2018). Impact of chocolate on the cardiovascular health. Front. Biosci..

[22] Godos J., Marventano S., Mistretta A., Galvano F., Grosso G. (2017). Dietary sources of polyphenols in the Mediterranean healthy Eating, Aging and Lifestyle (MEAL) study cohort. Int. J. Food Sci. Nutr..

[23] Fraga C.G., Croft K.D., Kennedy D.O., Tomás-Barberán F.A. (2019). The effects of polyphenols and other bioactives on human health. Food Funct..

[24] Katz J.M., Libman R.B., Wang J.J., Sanelli P., Filippi C.G., Gribko M., Pacia S.V., Kuzniecky R.I., Najjar S., Azhar S. (2020). Cerebrovascular Complications of COVID-19. Stroke.

[25] Chang W.-T., Toh H.S., Liao C.-T., Yu W.-L. (2021). Cardiac Involvement of COVID-19: A Comprehensive Review. Am. J. Med. Sci..

[26] Long B., Brady W.J., Koyfman A., Gottlieb M. (2020). Cardiovascular complications in COVID-19. Am. J. Emerg. Med..

[27] Clerkin K.J., Fried J.A., Raikhelkar J., Sayer G., Griffin J.M., Masoumi A., Jain S.S., Burkhoff D., Kumaraiah D., Rabbani L.R. (2020). COVID-19 and Cardiovascular Disease. Circulation.

[28] Guo T., Fan Y., Chen M., Wu X., Zhang L., He T., Wang H., Wan J., Wang X., Lu Z. (2020). Cardiovascular Implications of Fatal Outcomes of Patients With Coronavirus Disease 2019 (COVID-19). JAMA Cardiol..

[29] Gordon A.C., Mouncey P.R., Al-Beidh F., Rowan K.M., Nichol A.D., Arabi Y.M., Annane D., Beane A., Van Bentum-Puijk W., Berry L.R. (2021). Interleukin-6 Receptor Antagonists in Critically Ill Patients with Covid-19. N. Engl. J. Med..

[30] Aggarwal G., Cheruiyot I., Aggarwal S., Wong J., Lippi G., Lavie C.J., Henry B.M., Sanchis-Gomar F. (2020). Association of Cardiovascular Disease With Coronavirus Disease 2019 (COVID-19) Severity: A Meta-Analysis. Curr. Probl. Cardiol..

[31] Koopmann A., Georgiadou E., Reinhard I., Müller A., Lemenager T., Kiefer F., Hillemacher T. (2021). The Effects of the Lockdown during the COVID-19 Pandemic on Alcohol and Tobacco Consumption Behavior in Germany. Eur. Addict. Res..

[32] Li G., Saguner A.M., An J., Ning Y., Day J.D., Ding L., Waintraub X., Wang J. (2020). Cardiovascular disease during the covid-19 pandemic: Think ahead, protect hearts, reduce mortality. Cardiol. J..

[33] Scarmozzino F., Visioli F. (2020). Covid-19 and the subsequent lockdown modified dietary habits of almost half the population in an Italian sample. Foods.

[34] Ma Y., Ratnasabapathya R., Gardiner J. (2018). Carbohydrate Craving- not everything is sweet. Curr. Opin. Clin. Nutr. Metab..

[35] Ren Y., Liu Y., Sun X.Z., Wang B.Y., Zhao Y., Liu D.C., Zhang D.D., Liu X.J., Zhang R.Y., Sun H.H. (2019). Chocolate consumption and risk of cardiovascular diseases: A meta-Analysis of prospective studies. Heart.

[36] Morze J., Schwedhelm C., Bencic A., Hoffmann G., Boeing H., Przybylowicz K., Schwingshackl L. (2020). Chocolate and risk of chronic disease: A systematic review and dose-response meta-analysis. Eur. J. Nutr..

[37] Tan T.Y.C., Lim X.Y., Yeo J.H.H., Lee S.W.H., Lai N.M. (2021). The Health Effects of Chocolate and Cocoa: A Systematic Review. Nutrients.

[38] Crescente M., Jessen G., Momi S., Höltje H.D., Gresele P., Cerletti C., De Gaetano G. (2009). Interactions of gallic acid, resveratrol, quercetin and aspirin at the platelet cyclooxygenase-1 level: Functional and modelling studies. Thromb. Haemost..

[39] Alakhali K.M., Vigneshwaran E., Shaik M.A.A. (2018). Effect of food and antacid on simvastatin bioavailability on healthy adult volunteers. J. Health Res. Rev..

[40] Zubair M.H., Zubair M.H., Zubair M.N., Zubair M.M., Aftab T., Asad F. (2011). Augmentation of anti-platelet effects of aspirin by chocolate. J. Pak. Med. Assoc..

[41] Collyer T.C., Gray D.J., Sandhu R., Berridge J., Lyons G. (2009). Assessment of platelet inhibition secondary to clopidogrel and aspirin therapy in preoperative acute surgical patients measured by Thrombelastography^®^ Platelet Mapping^TM^. Br. J. Anaesth..

[42] Koziolek M., Alcaro S., Augustijns P., Basit A.W., Grimm M., Hens B., Hoad C.L., Jedamzik P., Madla C.M., Maliepaard M. (2019). The mechanisms of pharmacokinetic food-drug interactions—A perspective from the UNGAP group. Eur. J. Pharm. Sci..

[43] Climent E., Benaiges D., Pedro-Botet J. (2021). Hydrophilic or Lipophilic Statins?. Front. Cardiovasc. Med..

[44] Palleria C., Di Paolo A., Giofrè C., Caglioti C., Leuzzi G., Siniscalchi A., De Sarro G., Gallelli L. (2013). Pharmacokinetic drug-drug interaction and their implication in clinical management. J. Res. Med. Sci..

[45] Virani S.S., Alonso A., Aparicio H.J., Benjamin E.J., Bittencourt M.S., Callaway C.W., Carson A.P., Chamberlain A.M., Cheng S., Delling F.N. (2021). Heart Disease and Stroke Statistics-2021 Update A Report from the American Heart Association. Circulation.

[46] Lin X., Zhang I., Li A., Manson J.A.E., Sesso H.D., Wang L., Liu S. (2016). Cocoa flavanol intake and biomarkers for cardiometabolic health: A systematic review and meta-analysis of randomized controlled trials. J. Nutr..

[47] Folsom A.R., Chambless L.E., Ballantyne C.M., Coresh J., Heiss G., Wu K.K., Boerwinkle E., Mosley T.H., Sorlie P., Diao G. (2006). An assessment of incremental coronary risk prediction using C-reactive protein and other novel risk markers: The atherosclerosis risk in communities study. Arch. Intern. Med..

[48] Rodondi N., Marques-Vidal P., Butler J., Sutton-Tyrrell K., Cornuz J., Satterfield S., Harris T., Bauer D.C., Ferrucci L., Vittinghoff E. (2010). Markers of atherosclerosis and inflammation for prediction of coronary heart disease in older adults. Am. J. Epidemiol..

[49] Tibaut M., Caprnda M., Kubatka P., Sinkovič A., Valentova V., Filipova S., Gazdikova K., Gaspar L., Mozos I., Egom E.E. (2019). Markers of Atherosclerosis: Part 1—Serological Markers. Hear. Lung Circ..

[50] Bonnefont-Rousselot D. (2016). Resveratrol and cardiovascular diseases. Nutrients.

[51] Vázquez-Agell M., Sacanella E., Tobias E., Monagas M., Antúnez E., Zamora-Ros R., Andrés-Lacueva C., Lamuela-Raventós R.M., Fernández-Solá J., Nicolás J.M. (2007). Inflammatory markers of atherosclerosis are decreased after moderate consumption of cava (sparkling wine) in men with low cardiovascular risk. J. Nutr..

[52] Stockley C.S. (2016). The relationships between alcohol, wine and cardiovascular diseases—A review. Nutr. Aging.

[53] Roth I., Casas R., Ribó-Coll M., Doménech M., Lamuela-Raventós R.M., Estruch R. (2019). Acute consumption of Andalusian aged wine and gin decreases the expression of genes related to atherosclerosis in men with high cardiovascular risk: Randomized intervention trial. Clin. Nutr..

[54] Boronat A., Mateus J., Soldevila-Domenech N., Guerra M., Rodríguez-Morató J., Varon C., Muñoz D., Barbosa F., Morales J.C., Gaedigk A. (2019). Cardiovascular benefits of tyrosol and its endogenous conversion into hydroxytyrosol in humans. A randomized, controlled trial. Free Radic. Biol. Med..

[55] Da Costa Teixeira A.M.N., Luzia L.A., de Souza S.J., de Almeida Petrilli A., De Moraes Pontilho P., de Souza J.M.P., Segurado A.A.C., Efraim P., De Melo Picone C., De Carvalho Rondo P.H. (2017). The impact of dark chocolate intake on arterial elasticity in individuals with HIV/AIDS undergoing ART: A randomized, double-blind, crossover trial. Food Funct..

[56] Halib H., Ismail A., Mohd Yusof B.N., Osakabe N., Daud Z.A.M. (2020). Effects of cocoa polyphenols and dark chocolate on obese adults: A scoping review. Nutrients.

[57] Chiva-Blanch G., Urpi-Sarda M., Llorach R., Rotches-Ribalta M., Guillén M., Casas R., Arranz S., Valderas-Martinez P., Portoles O., Corella D. (2012). Differential effects of polyphenols and alcohol of red wine on the expression of adhesion molecules and inflammatory cytokines related to atherosclerosis: A randomized clinical trial (American Journal of Clinical Nutrition (2012) 95, (326–334)). Am. J. Clin. Nutr..

[58] Chiva-Blanch G., Urpi-Sarda M., Ros E., Valderas-Martinez P., Casas R., Arranz S., Guillén M., Lamuela-Raventós R.M., Llorach R., Andres-Lacueva C. (2013). Effects of red wine polyphenols and alcohol on glucose metabolism and the lipid profile: A randomized clinical trial. Clin. Nutr..

[59] Casas R., Estruch R., Sacanella E. (2018). Influence of bioactive nutrients on the atherosclerotic process: A review. Nutrients.

[60] Debreceni B., Debreceni L. (2014). Role of vitamins in cardiovascular health and disease. Res. Reports Clin. Cardiol..

[61] Imhof A., Blagieva R., Marx N., Koenig W. (2008). Drinking modulates monocyte migration in healthy subjects: A randomised intervention study of water, ethanol, red wine and beer with or without alcohol. Diabetes Vasc. Dis. Res..

[62] Giglio R.V., Patti A.M., Cicero A.F.G., Lippi G., Rizzo M., Toth P.P., Banach M. (2018). Polyphenols: Potential Use in the Prevention and Treatment of Cardiovascular Diseases. Curr. Pharm. Des..

[63] Lapuente M., Estruch R., Shahbaz M., Casas R. (2019). Relation of fruits and vegetables with major cardiometabolic risk factors, markers of oxidation, and inflammation. Nutrients.

[64] Curtis P.J., Dhatariya K., Sampson M., Kroon P.A., Potter J., Cassidy A. (2012). Chronic ingestion of flavan-3-ols and isoflavones improves insulin sensitivity and lipoprotein status and attenuates estimated 10-year CVD risk in medicated postmenopausal women with type 2 diabetes: A 1-year, double-blind, randomized, controlled trial. Diabetes Care.

[65] Ostertag L.M., Philo M., Colquhoun I.J., Tapp H.S., Saha S., Duthie G.G., Kemsley E.K., De Roos B., Kroon P.A., Le Gall G. (2017). Acute Consumption of Flavan-3-ol-Enriched Dark Chocolate Affects Human Endogenous Metabolism. J. Proteome Res..

[66] Nguyen C., Savouret J.F., Widerak M., Corvol M.T., Rannou F. (2017). Resveratrol, potential therapeutic interest in joint disorders: A critical narrative review. Nutrients.

[67] Kwok C.S., Loke Y.K., Welch A.A., Luben R.N., Lentjes M.A.H., Boekholdt S.M., Pfister R., Mamas M.A., Wareham N.J., Khaw K.T. (2016). Habitual chocolate consumption and the risk of incident heart failure among healthy men and women. Nutr. Metab. Cardiovasc. Dis..

[68] Veronese N., Demurtas J., Celotto S., Caruso M.G., Maggi S., Bolzetta F., Firth J., Smith L., Schofield P., Koyanagi A. (2019). Is chocolate consumption associated with health outcomes? An umbrella review of systematic reviews and meta-analyses. Clin. Nutr..

[69] Sun Y., Zimmermann D., De Castro C.A., Actis-Goretta L. (2019). Dose-response relationship between cocoa flavanols and human endothelial function: A systematic review and meta-analysis of randomized trials. Food Funct..

[70] Martini D., Rosi A., Tassotti M., Antonini M., Dall’Asta M., Bresciani L., Fantuzzi F., Spigoni V., Domínguez-Perles R., Angelino D. (2021). Effect of coffee and cocoa-based confectionery containing coffee on markers of cardiometabolic health: Results from the pocket-4-life project. Eur. J. Nutr..

[71] Mangels D.R., Mohler E.R. (2017). Catechins as potential mediators of cardiovascular health. Arterioscler. Thromb. Vasc. Biol..

[72] Regecova V., Jurkovicova J., Babjakova J., Bernatova I. (2020). The Effect of a Single Dose of Dark Chocolate on Cardiovascular Parameters and Their Reactivity to Mental Stress. J. Am. Coll. Nutr..

[73] Gianfredi V., Salvatori T., Nucci D., Villarini M., Moretti M. (2018). Can chocolate consumption reduce cardio-cerebrovascular risk? A systematic review and meta-analysis. Nutrition.

[74] Del Prete M., Samoggia A. (2020). Chocolate consumption and purchasing behaviour review: Research issues and insights for future research. Sustainability.

[75] Febrianto N.A., Wang S., Zhu F. (2021). Chemical and biological properties of cocoa beans affected by processing: A review. Crit. Rev. Food Sci. Nutr..

[76] Davinelli S., Corbi G., Righetti S., Sears B., Olarte H.H., Grassi D., Scapagnini G. (2018). Cardioprotection by cocoa polyphenols and ω-3 fatty acids: A disease-prevention perspective on aging-associated cardiovascular risk. J. Med. Food.

[77] Montagna M.T., Diella G., Triggiano F., Caponio G.R., De Giglio O., Caggiano G., Di Ciaula A., Portincasa P. (2019). Chocolate, “food of the gods”: History, science, and human health. Int. J. Environ. Res. Public Health.

[78] Kunachowicz H., Przygoda B., Nadolna I., Iwanow K. (2020). Food Composition Tables.

[79] Bandeira M.D., Maciel L.F., Bispo E.D., Soares S.E., MELOCW, SOUZACO (2020). Chemical composition and fatty acids profile of chocolates produced with different cocoa (*Theobroma cacao* L.) cultivars. Food Sci. Technol..

[80] Kühn J., Schröter A., Hartmann B.M., Stangl G.I. (2018). Cocoa and chocolate are sources of vitamin D 2. Food Chem..

[81] Okamoto T., Kobayashi R., Natsume M., Nakazato K. (2016). Habitual cocoa intake reduces arterial stiffness in postmenopausal women regardless of intake frequency: A randomized parallel-group study. Clin. Interv. Aging.

[82] Dala-Paula B.M., Deus V.L., Tavano O.L., Gloria M.B.A. (2021). In vitro bioaccessibility of amino acids and bioactive amines in 70% cocoa dark chocolate: What you eat and what you get. Food Chem..

[83] Oak M.H., Auger C., Belcastro E., Park S.H., Lee H.H., Schini-Kerth V.B. (2018). Potential mechanisms underlying cardiovascular protection by polyphenols: Role of the endothelium. Free Radic. Biol. Med..

[84] Talbot C.P.J.J., Mensink R.P., Smolders L., Bakeroot V., Plat J. (2018). Theobromine Does Not Affect Fasting and Postprandial HDL Cholesterol Efflux Capacity, While It Decreases Fasting miR-92a Levels in Humans. Mol. Nutr. Food Res..

[85] Montagnana M., Danese E., Salvagno G.L., Lippi G. (2017). Short-term effect of dark chocolate consumption on routine haemostasis testing. Int. J. Food Sci. Nutr..

[86] Neveu V., Perez-Jiménez J., Vos F., Crespy V., du Chaffaut L., Mennen L., Knox C., Eisner R., Cruz J., Wishart D. (2010). Phenol-Explorer: An online comprehensive database on polyphenol contents in foods. Database.

[87] Dong J.Y., Iso H., Yamagishi K., Sawada N., Tsugane S. (2017). Chocolate consumption and risk of stroke among men and women: A large population-based, prospective cohort study. Atherosclerosis.

[88] Latif R., Majeed F. (2020). Association between chocolate consumption frequency and heart rate variability indices. Explore.

[89] Vlachojannis J., Erne P., Zimmermann B., Chrubasik-Hausmann S. (2016). The Impact of Cocoa Flavanols on Cardiovascular Health. Phyther. Res..

[90] Steinhaus D.A., Mostofsky E., Levitan E.B., Dorans K.S., Håkansson N., Wolk A., Mittleman M.A. (2017). Chocolate intake and incidence of heart failure: Findings from the Cohort of Swedish Men. Am. Heart J..

[91] Mostofsky E., Berg Johansen M., Tjønneland A., Chahal H.S., Mittleman M.A., Overvad K. (2017). Chocolate intake and risk of clinically apparent atrial fibrillation: The Danish Diet, Cancer, and Health Study. Heart.

[92] Larsson S.C., Drca N., Jensen-Urstad M., Wolk A. (2018). Chocolate consumption and risk of atrial fibrillation: Two cohort studies and a meta-analysis. Am. Heart J..

[93] Jacob L., Smith L., Armstrong N.C., Yakkundi A., Barnett Y., Butler L., McDermott D.T., Koyanagi A., Shin J., Meyer J. (2021). Alcohol use and mental health during COVID-19 lockdown: A cross-sectional study in a sample of UK adults. Drug Alcohol Depend..

[94] O’Neil C.E., Fulgoni V.L., Nicklas T.A. (2011). Candy consumption was not associated with body weight measures, risk factors for cardiovascular disease, or metabolic syndrome in US adults: NHANES 1999–2004. Nutr. Res..

[95] Hammer A., Koppensteiner R., Steiner S., Niessner A., Goliasch G., Gschwandtner M., Hoke M. (2015). Dark chocolate and vascular function in patients with peripheral artery disease: A randomized, controlled cross-over trial. Clin. Hemorheol. Microcirc..

[96] Crichton G.E., Elias M.F., Dearborn P., Robbins M. (2017). Habitual chocolate intake and type 2 diabetes mellitus in the Maine-Syracuse Longitudinal Study: (1975–2010): Prospective observations. Appetite.

[97] Chareonrungrueangchai K., Wongkawinwoot K., Anothaisintawee T., Reutrakul S. (2020). Dietary factors and risks of cardiovascular diseases: An umbrella review. Nutrients.

[98] Ludovici V., Barthelmes J., Nägele M.P., Enseleit F., Ferri C., Flammer A.J., Ruschitzka F., Sudano I. (2017). Cocoa, Blood Pressure, and Vascular Function. Front. Nutr..

[99] Yuan S., Li X., Jin Y., Lu J. (2017). Chocolate consumption and risk of coronary heart disease, stroke, and diabetes: A meta-analysis of prospective studies. Nutrients.

[100] Mellor D.D., Georgousopoulou E.N., Naumovski N. (2017). Cocoa and chocolate, their clinical benefits: Insights in study design. CAB Rev. Perspect. Agric. Vet. Sci. Nutr. Nat. Resour..

[101] Fragopoulou E., Antonopoulou S. (2020). The French paradox three decades later: Role of inflammation and thrombosis. Clin. Chim. Acta.

[102] Mendonça R.D., Carvalho N.C., Martin-Moreno J.M., Pimenta A.M., Lopes A.C.S., Gea A., Martinez-Gonzalez M.A., Bes-Rastrollo M. (2019). Total polyphenol intake, polyphenol subtypes and incidence of cardiovascular disease: The SUN cohort study. Nutr. Metab. Cardiovasc. Dis..

[103] Vieira Humia B., Santos K.S., Mendonça Barbosa A., Sawata M., Da Costa Mendonça M., Ferreira Padilha F., Cosmi F., Di Giulio P., Masson S., Finzi A. (2016). The relationships between alcohol, wine and cardiovascular diseases—A review. Nutr. Metab. Cardiovasc. Dis..

[104] Gao M., Jebb S.A., Aveyard P., Ambrosini G.L., Perez-Cornago A., Carter J., Sun X., Piernas C. (2021). Associations between dietary patterns and the incidence of total and fatal cardiovascular disease and all-cause mortality in 116,806 individuals from the UK Biobank: A prospective cohort study. BMC Med..

[105] Mozaffarian D., Wu J.H.Y. (2018). Flavonoids, Dairy Foods, and Cardiovascular and Metabolic Health. Circ. Res..

[106] Almoosawi S., Tsang C., Ostertag L.M., Fyfe L., Al-Dujaili E.A.S. (2012). Differential effect of polyphenol-rich dark chocolate on biomarkers of glucose metabolism and cardiovascular risk factors in healthy, overweight and obese subjects: A randomized clinical trial. Food Funct..

[107] Kuebler U., Arpagaus A., Meister R.E., von Känel R., Huber S., Ehlert U., Wirtz P.H. (2016). Dark chocolate attenuates intracellular pro-inflammatory reactivity to acute psychosocial stress in men: A randomized controlled trial. Brain. Behav. Immun..

[108] Dower J.I., Geleijnse J.M., Kroon P.A., Philo M., Mensink M., Kromhout D., Hollman P.C.H. (2016). Does epicatechin contribute to the acute vascular function effects of dark chocolate? A randomized, crossover study. Mol. Nutr. Food Res..

[109] Cavarretta E., Peruzzi M., Del Vescovo R., Di Pilla F., Gobbi G., Serdoz A., Ferrara R., Schirone L., Sciarretta S., Nocella C. (2018). Dark chocolate intake positively modulates redox status and markers of muscular damage in elite football athletes: A randomized controlled study. Oxid. Med. Cell. Longev..

[110] Lee Y., Berryman C.E., West S.G., Chen C.Y.O., Blumberg J.B., Lapsley K.G., Preston A.G., Fleming J.A., Kris-Etherton P.M., Kris-Etherton P.M. (2017). Effects of Dark Chocolate and Almonds on Cardiovascular Risk Factors in Overweight and Obese Individuals: A Randomized Controlled-Feeding Trial. J. Am. Heart Assoc..

[111] Pereira T., Bergqvist J., Vieira C., Grüner Sveälv B., Castanheira J., Conde J. (2019). Randomized study of the effects of cocoa-rich chocolate on the ventricle–arterial coupling and vascular function of young, healthy adults. Nutrition.

[112] Barrios M., Orozco L.C., Stashenko E.E. (2018). Cocoa ingestion protects plasma lipids in healthy males against ex vivo oxidative conditions: A randomized clinical trial. Clin. Nutr. ESPEN.

[113] Loffredo L., Baratta F., Ludovica P., Battaglia S., Carnevale R., Nocella C., Novo M., Pannitteri G., Ceci F., Angelico F. (2018). Effects of dark chocolate on endothelial function in patients with non-alcoholic steatohepatitis. Nutr. Metab. Cardiovasc. Dis..

[114] Hurtado-Barroso S., Quifer-Rada P., de Alvarenga J.F.R., Pérez-Fernández S., Tresserra-Rimbau A., Lamuela-Raventos R.M. (2018). Changing to a low-polyphenol diet alters vascular biomarkers in healthy men after only two weeks. Nutrients.

[115] Bertolami A., Botelho P.B., Macedo L.F.L., Abdalla D.S.P., Faludi A.A., Galasso M., Castro I. (2014). Pleiotropic effects of plant sterols compared with ezetimibe on atherosclerosis biomarkers in type 2 diabetic patients treated with statins. Atherosclerosis.

[116] Mach F., Baigent C., Catapano A.L., Koskinas K.C., Casula M., Badimon L., Chapman M.J., De Backer G.G., Delgado V., Ference B.A. (2020). 2019 ESC/EAS Guidelines for the management of dyslipidaemias: Lipid modification to reduce cardiovascular risk. Eur. Heart J..

[117] Haseeb S., Alexander B., Santi R.L., Liprandi A.S., Baranchuk A. (2019). What’s in wine? A clinician’s perspective. Trends Cardiovasc. Med..

[118] Cuesta A., Haseeb S., Aquistapache F., Grosso P., Alexander B., Hopman W., Santi R.L., Baranchuk A. (2019). Alcohol consumption and cardiovascular health: A nationwide survey of Uruguayan cardiologists. Alcohol.

[119] Radonjić S., Maraš V., Raičević J., Košmerl T. (2020). Wine or beer? Comparison, changes and improvement of polyphenolic compounds during technological phases. Molecules.

[120] International Organisation of Vine and Wine (2020). OIV State of the World Vitivinicultural Sector in 2019.

[121] Arranz S., Chiva-Blanch G., Valderas-Martínez P., Medina-Remón A., Lamuela-Raventós R.M., Estruch R. (2012). Wine, beer, alcohol and polyphenols on cardiovascular disease and cancer. Nutrients.

[122] Pizzi A. (2021). Tannins medical/pharmacological and related applications: A critical review. Sustain. Chem. Pharm..

[123] Markoski M.M., Garavaglia J., Oliveira A., Olivaes J., Marcadenti A. (2016). Molecular properties of red wine compounds and cardiometabolic benefits. Nutr. Metab. Insights.

[124] Santhakumar A.B., Battino M., Alvarez-Suarez J.M. (2018). Dietary polyphenols: Structures, bioavailability and protective effects against atherosclerosis. Food Chem. Toxicol..

[125] Jacobs D.M., Fuhrmann J.C., Van Dorsten F.A., Rein D., Peters S., Van Velzen E.J.J., Hollebrands B., Draijer R., Van Duynhoven J., Garczarek U. (2012). Impact of short-term intake of red wine and grape polyphenol extract on the human metabolome. J. Agric. Food Chem..

[126] Murillo A.G., Fernandez M.L. (2017). The Relevance of Dietary Polyphenols in Cardiovascular Protection. Curr. Pharm. Des..

[127] Tresserra-Rimbau A., Rimm E.B., Medina-Remón A., Martínez-González M.A., de la Torre R., Corella D., Salas-Salvadó J., Gómez-Gracia E., Lapetra J., Arós F. (2014). Inverse association between habitual polyphenol intake and incidence of cardiovascular events in the PREDIMED study. Nutr. Metab. Cardiovasc. Dis..

[128] Noad R.L., Rooney C., McCall D., Young I.S., McCance D., McKinley M.C., Woodside J.V., McKeown P.P. (2016). Beneficial effect of a polyphenol-rich diet on cardiovascular risk: A randomised control trial. Heart.

[129] Ribeiro T.P., Oliveira A.C., Mendes-Junior L.G., França K.C., Nakao L.S., Schini-Kerth V.B., Medeiros I.A. (2016). Cardiovascular effects induced by northeastern Brazilian red wine: Role of nitric oxide and redox sensitive pathways. J. Funct. Foods.

[130] Snopek L., Mlcek J., Sochorova L., Baron M., Hlavacova I., Jurikova T., Kizek R., Sedlackova E., Sochor J. (2018). Contribution of red wine consumption to human health protection. Molecules.

[131] Bennemann G.D., de Assis C.F., Moreira G.C.R.C., de Lima L.H., Carvalho K.K., Torres Y.R., Botelho R.V. (2016). Mineral analysis, anthocyanins and phenolic compounds in wine residues flour. BIO Web Conf..

[132] Soldevila-Domenech N., Boronat A., Mateus J., Diaz-Pellicer P., Matilla I., Pérez-Otero M., Aldea-Perona A., De La Torre R. (2019). Generation of the antioxidant hydroxytyrosol from tyrosol present in beer and red wine in a randomized clinical trial. Nutrients.

[133] Andersen G., Marcinek P., Sulzinger N., Schieberle P., Krautwurst D. (2019). Food sources and biomolecular targets of tyramine. Nutr. Rev..

[134] Di Lorenzo C., Badea M., Colombo F., Orgiu F., Frigerio G., Pastor R.F., Restani P. (2017). Antioxidant activity of wine assessed by different in vitro methods. BIO Web Conf..

[135] Tverdal A., Magnus P., Selmer R., Thelle D. (2017). Consumption of alcohol and cardiovascular disease mortality: A 16 year follow-up of 115,592 Norwegian men and women aged 40–44 years. Eur. J. Epidemiol..

[136] Ye J., Chen X., Bao L., Tarantino G. (2019). Effects of wine on blood pressure, glucose parameters, and lipid profile in type 2 diabetes mellitus: A meta-analysis of randomized interventional trials (PRISMA Compliant). Medicine.

[137] Révész D., Bours M.J.L., Wegdam J.A., Keulen E.T.P., Breukink S.O., Slooter G.D., Vogelaar F.J., Weijenberg M.P., Mols F. (2021). Associations between alcohol consumption and anxiety, depression, and health-related quality of life in colorectal cancer survivors. J. Cancer Surviv..

[138] Fernandes I., Pérez-Gregorio R., Soares S., Mateus N., De Freitas V., Santos-Buelga C., Feliciano A.S. (2017). Wine flavonoids in health and disease prevention. Molecules.

[139] Mankowski R.T., You L., Buford T.W., Leeuwenburgh C., Manini T.M., Schneider S., Qiu P., Anton S.D. (2020). Higher dose of resveratrol elevated cardiovascular disease risk biomarker levels in overweight older adults—A pilot study. Exp. Gerontol..

[140] McDermott M.M., Leeuwenburgh C., Guralnik J.M., Tian L., Sufit R., Zhao L., Criqui M.H., Kibbe M.R., Stein J.H., Lloyd-Jones D. (2017). Effect of resveratrol onwalking performance in older people with peripheral artery disease the restore randomized clinical trial. JAMA Cardiol..

[141] Fragopoulou E., Choleva M., Antonopoulou S., Demopoulos C.A. (2018). Wine and its metabolic effects. A comprehensive review of clinical trials. Metabolism..

[142] Nova E., San Mauro-Martín I., Díaz-Prieto L.E., Marcos A. (2019). Wine and beer within a moderate alcohol intake is associated with higher levels of HDL-c and adiponectin. Nutr. Res..

[143] Droste D.W., Iliescu C., Vaillant M., Gantenbein M., De Bremaeker N., Lieunard C., Velez T., Meyer M., Guth T., Kuemmerle A. (2014). Advice on lifestyle changes (Diet, Red Wine and Physical Activity) does not affect internal carotid and middle cerebral artery blood flow velocity in patients with carotid arteriosclerosis in a randomized controlled trial. Cerebrovasc. Dis..

[144] Eker M.E., Aaby K., Budic-Leto I., Brncic S.R., El S.N., Karakaya S., Simsek S., Manach C., Wiczkowski W., De Pascual-Teresa S. (2020). A review of factors affecting anthocyanin bioavailability: Possible implications for the inter-individual variability. Foods.

[145] Karadeniz M., Akçay Y.D., Yildirim H.K., Yilmaz C., Sözmen E.Y. (2014). Effect of red wine consumption on serum oxidation and adiponectin levels in overweight and healthy individuals. Pol. J. Food Nutr. Sci..

[146] Di Castelnuovo A., Costanzo S., Bonaccio M., Rago L., De Curtis A., Persichillo M., Bracone F., Olivieri M., Cerletti C., Donati M.B. (2017). Moderate Alcohol Consumption Is Associated With Lower Risk for Heart Failure But Not Atrial Fibrillation. JACC Hear. Fail..

[147] Da Luz P.L., Favarato D., Moriguchi E.H., De Carli W., Bruscato N., Mochiduky R.I., Schwartzman P., Rochitte C.E., Laurindo F.R. (2018). Red wine consumption, coronary calcification, and long-term clinical evolution. Braz. J. Med. Biol. Res..

[148] Panagiotakos D.B., Kouli G.M., Magriplis E., Kyrou I., Georgousopoulou E.N., Chrysohoou C., Tsigos C., Tousoulis D., Pitsavos C. (2019). Beer, wine consumption, and 10-year CVD incidence: The ATTICA study. Eur. J. Clin. Nutr..

[149] Park H., Kim K. (2012). Association of alcohol consumption with lipid profile in hypertensive men. Alcohol Alcohol..

[150] Attard R., Dingli P., Doggen C.J.M.M., Cassar K., Farrugia R., Bezzina Wettinger S. (2021). The impact of frequency, pattern, intensity, and type of alcohol consumption, and its combined effect with smoking on inflammation, lipid profile, and the risk of myocardial infarction. J. Public Health.

[151] Ruf J.C. (2004). Alcohol, wine and platelet function. Biol. Res..

[152] Levantesi G., Marfisi R., Mozaffarian D., Franzosi M.G., Maggioni A., Nicolosi G.L., Schweiger C., Silletta M., Tavazzi L., Tognoni G. (2013). Wine consumption and risk of cardiovascular events after myocardial infarction: Results from the GISSI-Prevenzione trial. Int. J. Cardiol..

[153] Jani B.D., McQueenie R., Nicholl B.I., Field R., Hanlon P., Gallacher K.I., Mair F.S., Lewsey J. (2021). Association between patterns of alcohol consumption (beverage type, frequency and consumption with food) and risk of adverse health outcomes: A prospective cohort study. BMC Med..

[154] Gepner Y., Golan R., Harman-Boehm I., Henkin Y., Schwarzfuchs D., Shelef I., Durst R., Kovsan J., Bolotin A., Leitersdorf E. (2015). Effects of initiating moderate alcohol intake on cardiometabolic risk in adults with type 2 diabetes: A 2-year randomized, controlled trial. Ann. Intern. Med..

[155] Droste D.W., Iliescu C., Vaillant M., Gantenbein M., De Bremaeker N., Lieunard C., Velez T., Meyer M., Guth T., Kuemmerle A. (2013). A daily glass of red wine associated with lifestyle changes independently improves blood lipids in patients with carotid arteriosclerosis: Results from a randomized controlled trial. Nutr. J..

[156] Di Castelnuovo A., Costanzo S., Bonaccio M., McElduff P., Linneberg A., Salomaa V., Männistö S., Moitry M., Ferrières J., Dallongeville J. (2021). Alcohol Intake and Total Mortality in 142,960 Individuals from the MORGAM Project: A population-based study. Addiction.

[157] De La Torre R., Corella D., Castañer O., Martínez-González M.A., Salas-Salvador J., Vila J., Estruch R., Sorli J.V., Arós F., Fiol M. (2017). Protective effect of homovanillyl alcohol on cardiovascular disease and total mortality: Virgin olive oil, wine, and catechol-methylathion. Am. J. Clin. Nutr..

[158] Kechagias S., Zanjani S., Gjellan S., Leinhard O.D., Kihlberg J., Smedby Ö., Johansson L., Kullberg J., Ahlström H., Lindström T. (2011). Effects of moderate red wine consumption on liver fat and blood lipids: A prospective randomized study. Ann. Med..

[159] Taborsky M., Ostadal P., Adam T., Moravec O., Gloger V., Schee A., Skala T. (2017). Red or white wine consumption effect on atherosclerosis in healthy individuals (In Vino Veritas study). Taborsky. Bratislava Med. J..

[160] Huang P.H., Chen Y.H., Tsai H.Y., Chen J.S., Wu T.C., Lin F.Y., Sata M., Chen J.W., Lin S.J. (2010). Intake of red wine increases the number and functional capacity of circulating endothelial progenitor cells by enhancing nitric oxide bioavailability. Arterioscler. Thromb. Vasc. Biol..

[161] Do Rosario V.A., Schoenaker D.A.J.M.J.M., Kent K., Weston-Green K., Charlton K. (2020). Association between flavonoid intake and risk of hypertension in two cohorts of Australian women: A longitudinal study. Eur. J. Nutr..

[162] Boydens C., Pauwels B., Vanden Daele L., Van de Voorde J. (2016). Protective effect of resveratrol and quercetin on in vitro-induced diabetic mouse corpus cavernosum. Cardiovasc. Diabetol..

[163] Giacosa A., Barale R., Bavaresco L., Faliva M.A., Gerbi V., La Vecchia C., Negri E., Opizzi A., Perna S., Pezzotti M. (2016). Mediterranean Way of Drinking and Longevity. Crit. Rev. Food Sci. Nutr..

[164] Violi F., Loffredo L., Carnevale R., Pignatelli P., Pastori D. (2017). Atherothrombosis and Oxidative Stress: Mechanisms and Management in Elderly. Antioxidants Redox Signal..

[165] Guilford J.M., Pezzuto J.M. (2011). Wine and health: A review. Am. J. Enol. Vitic..

[166] Wotherspoon A., Elshahat S., McAlinden N., Dean K., Young I.S., Sharpe P.C., Blankenburg S., Patterson C.C., McKinley M.C., Evans A. (2020). Effect of Moderate Red Wine versus Vodka Consumption on Inflammatory Markers Related to Cardiovascular Disease Risk: A Randomized Crossover Study. J. Am. Coll. Nutr..

[167] Di Renzo L., Cioccoloni G., Salimei P.S., Ceravolo I., De Lorenzo A., Gratteri S. (2018). Alcoholic beverage and meal choices for the prevention of noncommunicable diseases: A randomized nutrigenomic trial. Oxid. Med. Cell. Longev..

[168] Golan R., Gepner Y., Shai I. (2019). Wine and Health—New Evidence. Eur. J. Clin. Nutr..

[169] Mori T.A., Burke V., Zilkens R.R., Hodgson J.M., Beilin L.J., Puddey I.B. (2016). The effects of alcohol on ambulatory blood pressure and other cardiovascular risk factors in type 2 diabetes: A randomized intervention. J. Hypertens..

[170] Tousoulis D., Ntarladimas I., Antoniades C., Vasiliadou C., Tentolouris C., Papageorgiou N., Latsios G., Stefanadis C. (2008). Acute effects of different alcoholic beverages on vascular endothelium, inflammatory markers and thrombosis fibrinolysis system. Clin. Nutr..

[171] Şanlier N., Gökcen B.B., Sezgin A.C. (2019). Health benefits of fermented foods. Crit. Rev. Food Sci. Nutr..

[172] Bae J.-S., Oh A.-R., Cha J.-Y. (2013). Regulation of Cholesterol Metabolism in Liver: Link to NAFLD and Impact of n-3 PUFAs. J. Lifestyle Med..

[173] Wang H., Blumberg J.B., Chen C.Y.O., Choi S.W., Corcoran M.P., Harris S.S., Jacques P.F., Kristo A.S., Lai C.Q., Lamon-Fava S. (2014). Dietary modulators of statin efficacy in cardiovascular disease and cognition. Mol. Aspects Med..

[174] Antal E.J., Hendershot P.E., Batts D.H. (2001). Linezolid, a novel oxazolidinone antibiotic: Assessment of monoamine oxidase inhibition using pressor response to oral tyramine. J. Clin. Pharmacol..

[175] Mergenhagen K.A., Wattengel B.A., Skelly M.K., Clark C.M., Russo T.A. (2020). Fact versus fiction: A review of the evidence behind alcohol and antibiotic interactions. Antimicrob. Agents Chemother..

[176] Flanagan S., Bartizal K., Minassian S.L., Fang E., Prokocimer P. (2013). In vitro, In Vivo, and clinical studies of tedizolid to assess the potential for peripheral or central monoamine oxidase interactions. Antimicrob. Agents Chemother..

[177] Scolaro B., Nogueira M.S., Paiva A., Bertolami A., Barroso L.P., Vaisar T., Heffron S.P., Fisher E.A., Castro I.A. (2018). Statin dose reduction with complementary diet therapy: A pilot study of personalized medicine. Mol. Metab..

[178] Piotrowicz J., Pazur A., Zachwieja Z. (2008). Statyny i ich interakcje z pożywieniem. Bromat. Chem. Toksykol..

[179] Smith H.T., Jokubaitis L.A., Troendle A.J., Hwang D.S., Robinson W.T. (1993). Pharmacokinetics of fluvastatin and specific drug interactions. Am. J. Hypertens..

[180] Won C., Oberlies N., Paine M. (2012). Mechanisms Underlying Food-Drug Interactions: Inhibition of Intestinal Metabolism and Transport Christina. Pharmacol. Ther..

[181] Hyrsova L., Vanduchova A., Dusek J., Smutny T., Carazo A., Maresova V., Trejtnar F., Barta P., Anzenbacher P., Dvorak Z. (2019). Trans-resveratrol, but not other natural stilbenes occurring in food, carries the risk of drug-food interaction via inhibition of cytochrome P450 enzymes or interaction with xenosensor receptors. Toxicol. Lett..

[182] Basheer L., Kerem Z. (2015). Interactions between CYP3A4 and Dietary Polyphenols. Oxid. Med. Cell. Longev..

[183] Detampel P., Beck M., Krähenbühl S., Huwyler J. (2012). Drug interaction potential of resveratrol. Drug Metab. Rev..

[184] Briguglio M., Hrelia S., Malaguti M., Serpe L., Canaparo R., Dell’osso B., Galentino R., De Michele S., Dina C.Z., Porta M. (2018). Food bioactive compounds and their interference in drug pharmacokinetic/pharmacodynamic profiles. Pharmaceutics.

